# Non-cyclic formylated dipyrromethanes as phosphate anion receptors†
†Electronic supplementary information (ESI) available: Synthetic details, NMR and UV-Vis spectroscopic data, electrochemical analyses, and X-ray diffraction data. CCDC 1444557–1444561. For ESI and crystallographic data in CIF or other electronic format see DOI: 10.1039/c6sc00015k


**DOI:** 10.1039/c6sc00015k

**Published:** 2016-02-24

**Authors:** Murat K. Deliomeroglu, Vincent M. Lynch, Jonathan L. Sessler

**Affiliations:** a Department of Chemistry , The University of Texas at Austin , Texas 78712-1224 , USA . Email: Sessler@cm.utexas.edu

## Abstract

New *tetrakis*- and *hexakis*(1*H*-pyrrole-2-carbaldehyde) anion receptors are described. The anion binding properties of these receptors were studied in organic media and in the solid state. The receptors displayed good affinity for the dihydrogenphosphate and pyrophosphate anions (as the tetrabutylammonium salts) in chloroform even in the presence of a polar protic solvent, methanol. Solution phase spectroscopic analyses proved consistent with the binding mode seen in single crystal structural studies of the dihydrogenphosphate and pyrophosphate complexes and provided support for the contention that these receptors undergo conformational reorganization in order to accommodate the bound oxoanions both in chloroform solution and in the solid state.

## Abstract 2

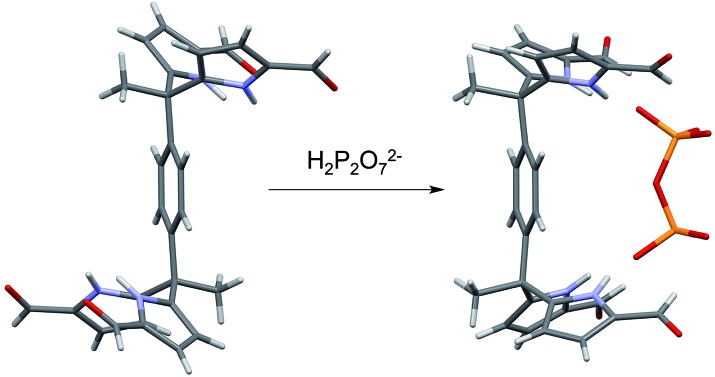
 *Tetrakis*- and *hexakis*(1*H*-pyrrole-2-carbaldehyde) anion receptors are described that undergo conformational reorganization in order to accommodate the dihydrogenphosphate and pyrophosphate anions.

## Introduction

**Scheme 1:**
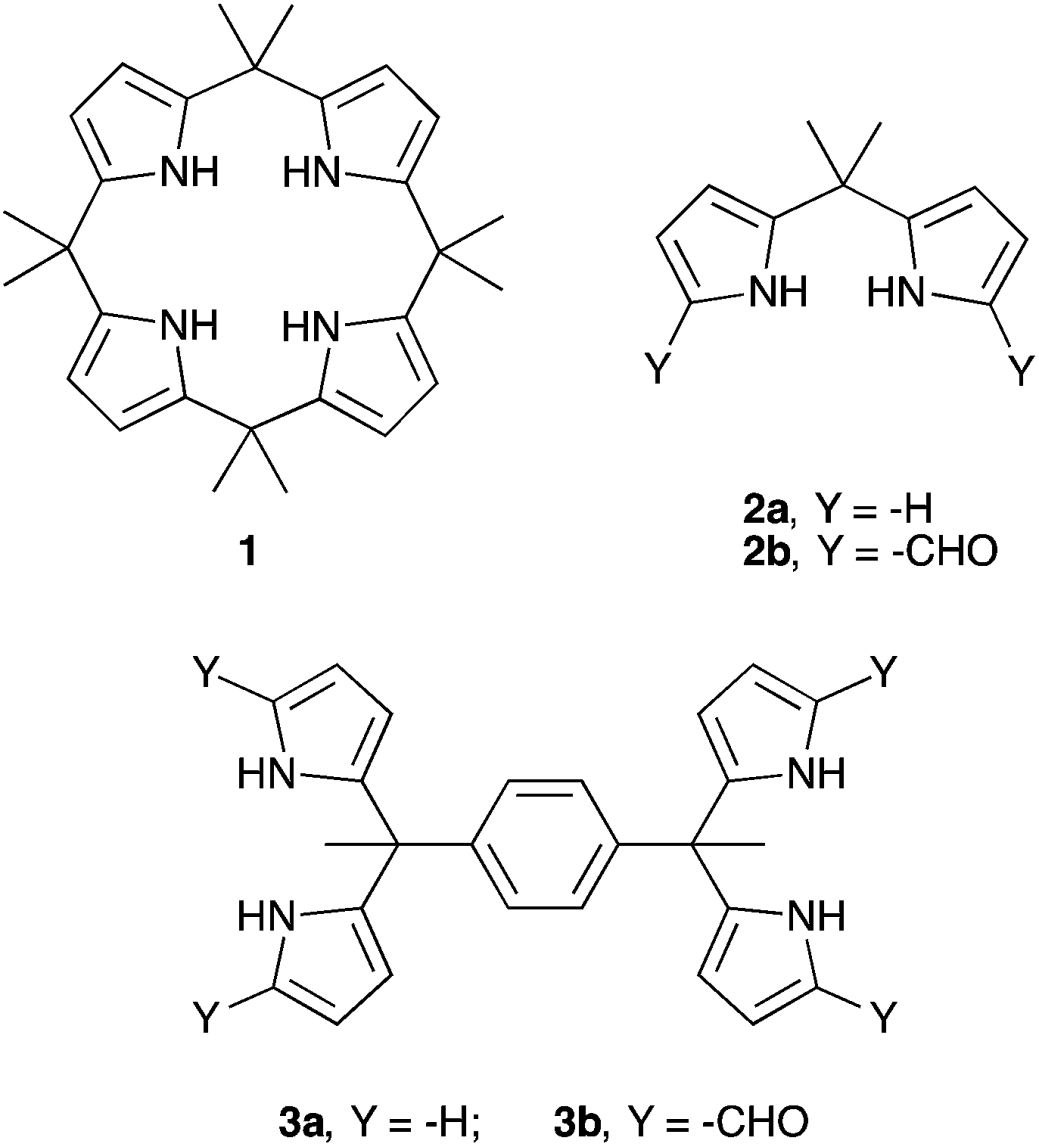
Structures of compounds **1**, **2a-b**, and **3a-b**.

Inorganic phosphate and related anions are ubiquitous in nature. These anionic species play key roles in energy transduction and in the storage and expression of genetic information.^1^ Phosphates are also pervasive pollutants and may lead to eutrophication of lakes and waterways.^2^ The development of molecular constructs capable of binding the phosphate anion in its various protonated forms, as well as related oxoanion species is of critical interest since it may provide a first step towards solving various problems related to anion detection, extraction, and separation.^3^–^6^ In general, anions are larger than cations and therefore larger hosts are required to bind them.^7^ Fortunately, a number of binding motifs may be exploited to achieve anion recognition, including coulombic interactions, hydrogen bonding, halogen bonding, and anion–π interactions.^8^–^12^ Hydrogen bonding has proven particularly effective. However, certain anions, including oxoanions, such as dihydrogenphosphate (H_2_PO_4_^–^) and hydrogensulfate (HSO_4_^–^), are susceptible to proton transfer.^13^ As a result, ensuring anion binding rather than deprotonation (acid–base chemistry) has proven to be a challenging task.^14^,^15^ One hydrogen bond donor that is relatively less susceptible to deprotonation is pyrrole. An early example of a pyrrole-based system capable of binding the phosphate anion was the pentapyrrolic macrocycle, sapphyrin. This system, which is dicationic in its protonated form, proved capable of stabilizing a “sitting-atop” complex with the phosphate anion in the solid state and of binding DNA and other phosphate-containing species in solution.^16^ In 1996, Gale *et al.* reported that octamethylcalix[4]pyrrole (C[4]P; **1**) may act as an anion receptor in organic media.^17^ Calix[4]pyrrole is a non-conjugated system long known in the literature.^18^ It was found to bind the fluoride and chloride anions in halogenated solvents, along with their counter cations in some cases.^19^,^20^ Halide anion recognition by C[4]P is accompanied by conformational change from a limiting 1,3-alternate conformation (the free form) to a cone conformation (halide-bonded form). This structural switching allows the formation of four NH–anion hydrogen bonds in the case of simple halide anions.^17^ Under most experimental conditions, C[4]P has proved less effective as a receptor for larger anions, including the dihydrogenphosphate anion. There thus remains a need for simple-to-prepare analogues of C[4]P that bind phosphate. Open-chain systems might allow this goal to be attained (Scheme 1).

In the case of fluoride and chloride anion salts studied in organic media, compound **1** displayed association constants that were >2× greater than those recorded for the corresponding acyclic building block, dipyrromethane (DPM) **2a**.^21^ This difference was ascribed to the greater pre-organization of the cyclic systems, as well as to the increased number of pyrrolic NH hydrogen bond donors provided by **1** compared to **2a**. Interestingly, compound **2a** displayed a higher affinity towards H_2_PO_4_^–^ (as its tetra-*n*-butylammonium (TBA^+^) salt) than did **1** in CD_2_Cl_2_. While this apparent dichotomy was not studied in detail, it might reflect the fact that the dihydrogenphosphate anion is too large to be accommodated well within the NH-rich calix[4]pyrrole pseudo cavity. In contrast, the presumably greater flexibility of **2a** might allow the stabilization of a greater number of favourable contacts with this and other oxoanions than compound **1**. While untested, this hypothesis provides an incentive to prepare and study new dipyrromethane-based anion receptors. A goal of this effort would be to create phosphate anion receptors that rival C[4]P in terms of ease of preparation and which allow for good phosphate anion binding. In this study we focus on dipyrromethane (DPM) receptors that have been subject to formylation in the so-called α-pyrrolic positions.

In 2003, in the context of synthetic work aimed at producing a C[4]P-texaphyrin chimera,^22^ the pyrrolic α-positions of **2a** were subject to formylation to produce **2b**. The anion binding properties of **2b** with TBAF were studied by isothermal calorimetry (ITC) in acetonitrile and *via* X-ray crystallography in the solid state.^23^ On this basis, it was concluded that **2b** was not an effective anion receptor. On the other hand, we recently found^24^ that the bisdipyyrromethane (bisDPM) **3a** and its formylated derivative **3b**, act as effective and somewhat selective receptors for the TBA^+^ salts of dihydrogenphosphate (H_2_PO_4_^–^) and hydrogenpyrophosphate (HP_2_O_7_^3–^) in chloroform, as inferred from NMR spectroscopic and UV-Vis analyses. As compared to **1**, the non-formylated system **3a** proved to be a slightly better receptor for H_2_PO_4_^–^ (*K*_a_ = 10^3^*vs.* 10^2^ M^–1^ in CHCl_3_ and CH_2_Cl_2_, respectively). DPM derivatives, which do not bear substituents in the pyrrolic α-positions are notoriously unstable, being prone *inter alia* to oxidative coupling and electrophilic aromatic substitution. On the other hand, bis-formylation yields stable systems that we believe may have a role to play as anion receptors. In preliminary work, high phosphate anion affinities were noted for the four-fold formylated derivative of **3a**, receptor **3b** (*K*_a_ = (8 ± 2) × 10^6^ M^–1^ for **3b***vs.* (1.0 ± 0.1) × 10^3^ M^–1^ for **3a** in CHCl_3_; *cf.*Table 1).^24^ Presumably, this increased affinity reflects the favourable electronic changes, as well as the addition of hydrogen bond accepting sites, that result from formylation. To understand the underlying determinants and to improve on the phosphate binding affinity displayed by **3b**, we have now prepared and studied several analogues bearing different spacer elements between the diformyl-DPM subunits, as well as a new *hexakis*(1*H*-pyrrole-2-carbaldehyde) receptor, **7b**. Compared to **3b**, receptor **7b** displays a phosphate anion affinity that is enhanced by roughly 66 fold when studied under identical conditions (*i.e.*, CHCl_3_ containing 3% CH_3_OH).

**Table 1:** Calculated H_2_PO_4_^–^ binding affinities, *K*_a_ (M^–1^) of dipyrromethane derivatives before (**3a** and **5a**) and after formylation (**3b** and **5b**)

**3a** ^*a*^ ^,^ ^*c*^	**3b** ^*b*^ ^,^ ^*c*^	**5a** ^*a*^	**5b** ^*b*^ ^,^ ^*c*^
(1.0 ± 0.1) × 10^3^	(8 ± 2) × 10^6^	40.8 ± 0.6	(4.6 ± 0.9) × 10^5^

^*a*^Determined using ^1^H NMR spectroscopy in CDCl_3_.

^*b*^Determined using UV-Vis spectroscopy in CHCl_3_.

^*c*^Values from ref. 24.

## Results and discussion

### Syntheses and single crystal structures

**Scheme 2:**
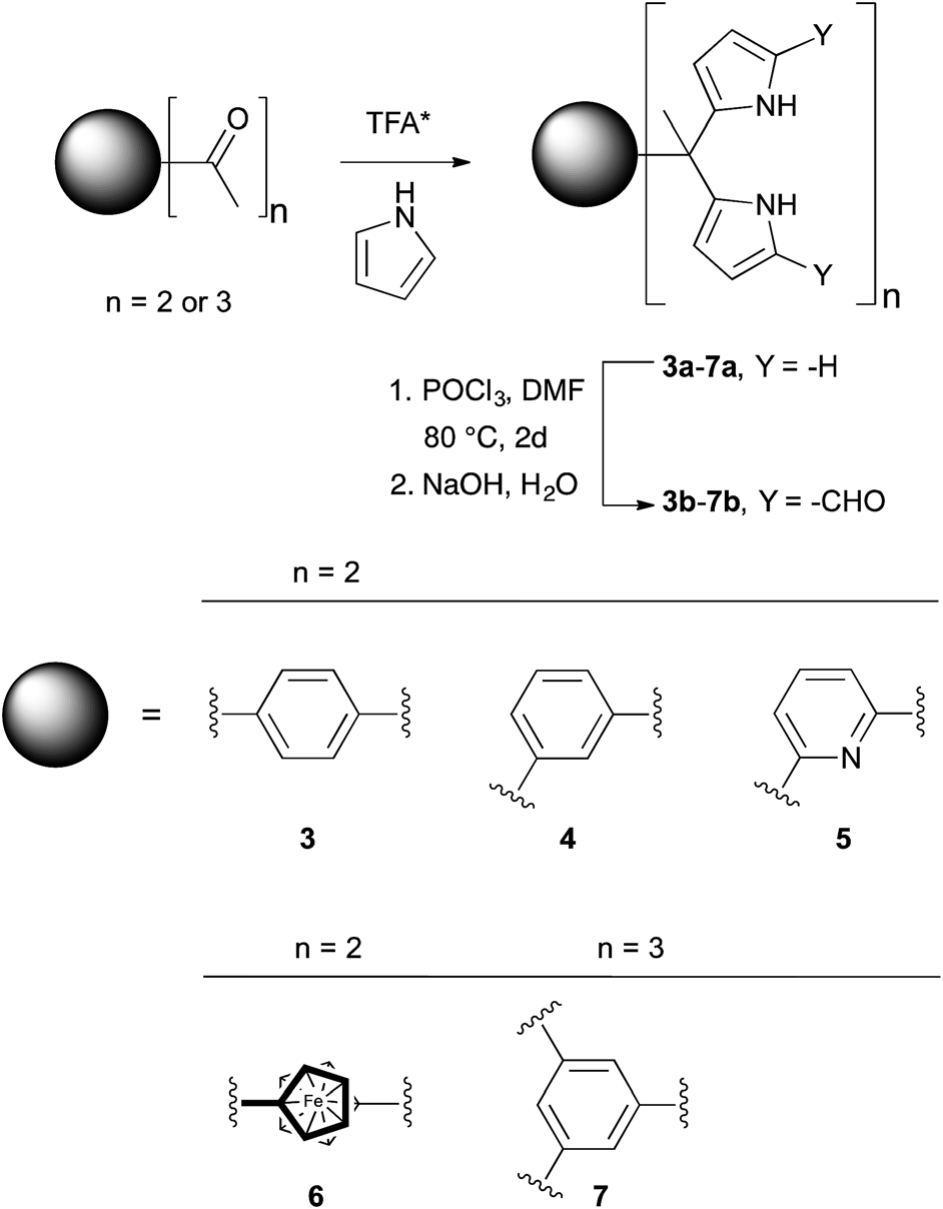
Synthesis of receptors **3b–7b**. *Catalyst concentrations and reaction temperatures vary.

The synthesis and characterization of **3b–5b** have been reported previously.^24^ In this paper, we present the new derivatives, **6b** and **7b**, obtained in moderate overall yields. The synthetic route to obtain **3b–7b** is shown in Scheme 2. In the first step, commercially available diacetyl and triacetyl starting materials were converted into bisDPM **6a**, and trisdipyrromethane (trisDPM) **7a**, respectively, albeit in low yields (5–15%). The low yields stand in contrast to high yields observed for **3a** and **5a**, which precipitate from the reaction mixture and constitute the dominant reaction products.^24^ The unstable α-free bisDPM (**6a**) and trisDPM (**7a**) were then subject to Vilsmeier–Haack formylation to produce the stable formylated products **6b** and **7b** in relatively high yields (*ca.* 80%). Purification of **6b** was carried out by recrystallization from *N*,*N*-dimethylformamide (DMF) at 80 °C, whereas **7b** was purified by column chromatography over silica gel. The molecular structures were characterized by ^1^H and ^13^C NMR spectroscopy, high-resolution mass spectrometry (HRMS), and single crystal X-ray diffraction analyses.

**Fig. 1:**
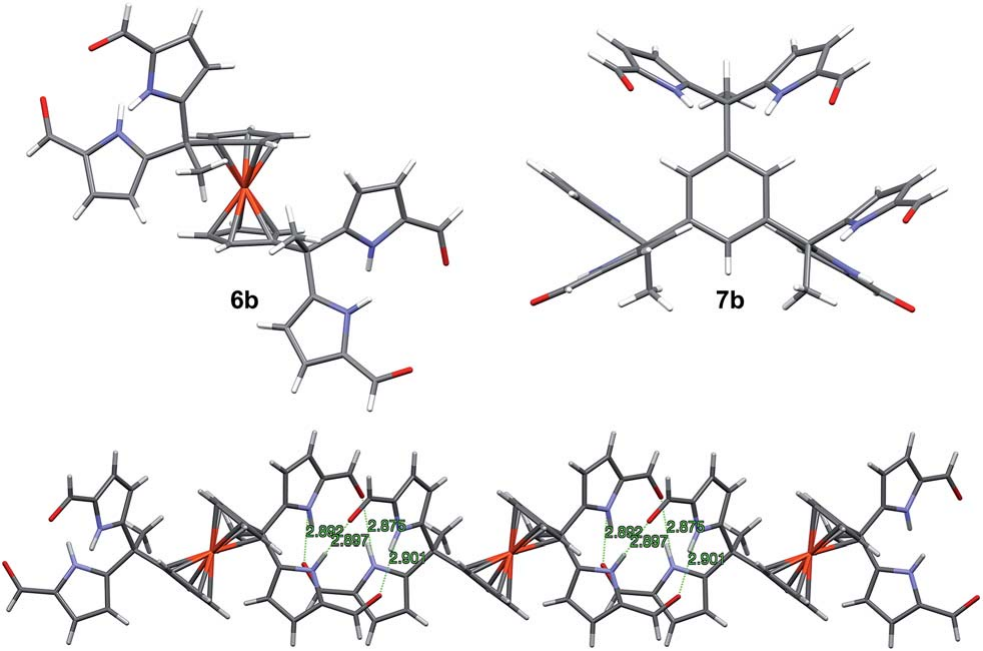
Top left: Single crystal structure of **6b** (DMF solvent molecules are omitted for clarity); Top right: Single crystal structure of **7b** (CHCl_3_ solvent molecules are omitted for clarity); Bottom: Linear assembly formed by one of the atropisomers of **6b**.

Receptor **6b** co-crystallised with two molecules of DMF. The single crystal structure revealed that two diformyl-DPM units residing on opposite cyclopentadienyl (Cp) rings of the ferrocene were locked at about 90° from one another, leading to the presence of a racemic mixture of conformational isomers within the single crystal. Fig. 1 shows one of the atropisomers of **6b**. It crystallizes as a one-dimensional hydrogen bond interlocked ensemble, even in the presence of DMF. The formation of these assemblies can be rationalized on a geometric basis. The bridging ferrocene adopts a conformation such that the two constituent Cp rings, each bearing a DPM substituent, are offset from one another by 90°. This allows for the formation of four intermolecular hydrogen bonds between the two self-complementary diformyl-DPM subunits, as can be seen from an inspection of Fig. 1.

Receptor **7b**, on the other hand, co-crystallised with CHCl_3_. Each of the three diformyl-DPM units on the bridging benzene is oriented in a different direction (*cf.*Fig. 1). One of the three DPM subunits was found to resemble what is seen in the case of **3b**^24^ wherein two diformyl-DPM units are seen to orient towards opposite π-faces of the intervening benzene ring. On the other hand, two of the diformyl-DPM units present in **7b** adopt conformations in which the pyrrole NH groups point away from one another. As a result, **7b** forms dimers stabilised by four hydrogen bonds that extend to create two-dimensional layers (*cf.* Fig. S2†). The assemblies formed by **6b** and **7b** may be viewed as being examples of Gale and co-worker's “narcissistic dimer”^15^ that are extended into one and two-dimensional space, respectively. The hydrogen bond interlocked aggregates seen in the solid state structures of **6b** and **7b** may account for the low solubility these species display in many solvents.

### Anion binding studies

The anion selectivity for **3b–5b** in CHCl_3_ was found to be H_2_PO_4_^–^ > HP_2_O_7_^3–^ > HSO_4_^–^ > C_6_H_5_CO_2_^–^ ≫ NO_3_^–^, Cl^–^ (as the commercially available TBA^+^ salts).^24^ The observed selectivity was not found to correlate with the basicity of the anions (basicity order: HP_2_O_7_^3–^ > C_6_H_5_CO_2_^–^ > H_2_PO_4_^–^ > HSO_4_^–^, NO_3_^–^, Cl^–^). Rather, the trend appears to correlate with an ability of the anion to interact with the receptor *via* hydrogen bond donation to the formyl groups (in addition to acting as a Lewis basic hydrogen bond acceptor for the pyrrolic NH protons). In fact, the H_2_PO_4_^–^ anion, with two hydrogen bond donating sites (rather than one or none as in the other test anions), is bound with the highest relative affinity.

The effect of the formyl groups in abetting dihydrogenphosphate anion binding is substantial (*i.e.*, several orders of magnitude difference in *K*_a_ values). This was first noted for **3a***vs.***3b** in the context of our original report (*vide supra*).^24^ Further support for this conclusion came from a comparison of the α-free derivative **5a** with its formylated congener **5b** (*cf.*Table 1). Unfortunately, the other non-formylated DPM derivatives prepared in the context of this study proved too unstable to allow for analyses of their anion binding affinities.

**Fig. 2:**
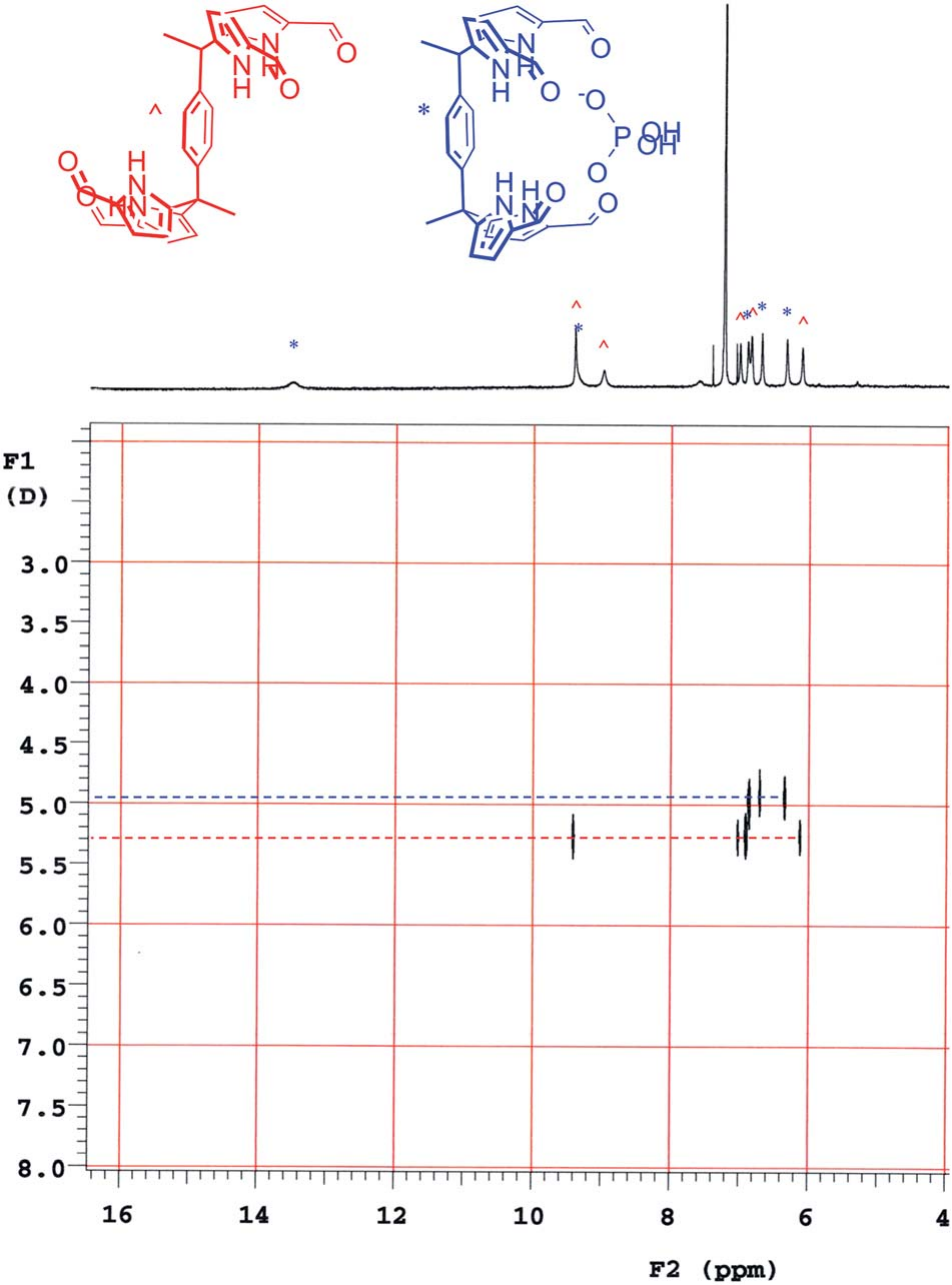
DOSY spectrum of a 1 : 0.5 mixture of **3b** and TBAH_2_PO_4_. The mixture was produced by titrating a 0.8 mM CDCl_3_ solution of TBAH_2_PO_4_ into a turbid mixture of **3b** in CDCl_3_ until the ratio of integration values corresponding to **3b** and **3b**·H_2_PO_4_^–^ was equal. ^ shows signals ascribed to free **3b**; * shows those assigned to **3b**·H_2_PO_4_^–^.

Diffusion ordered NMR spectroscopy (DOSY)^25^ was used to examine the interactions between anions and receptor **3b**. Receptor **3b** was chosen for these studies for two reasons. First, the low solubility of **3b** in CHCl_3_ means that an equilibrium was expected to exist between species free in solution and those tied up in the solid state. Secondly, on the ^1^H NMR time scale, free receptor **3b** and the H_2_PO_4_^–^ bound complex (**3b**·H_2_PO_4_^–^) were subject to slow-exchange,^24^ allowing both species to be observed concurrently. Fig. 2 shows the DOSY spectrum of a mixture of **3b** at the half-equivalence point obtained by titrating **3b** with TBAH_2_PO_4_ in CDCl_3_. The one-dimensional ^1^H NMR spectrum in the horizontal axis shows two sets of receptor signals; free **3b** and **3b**·H_2_PO_4_^–^, labelled with red and blue asterisks, respectively.

The two-dimensional spectrum is characterized by the presence of two sets of receptor signals corresponding to slightly different diffusion rates. The complex **3b**·H_2_PO_4_^–^ (blue) features a lower mobility than the free receptor **3b** (red). No evidence of higher order (*i.e.*, aggregated) species is seen. Based on these observations, we conclude that in the absence of complete conversion to the bound form, samples of **3b** and TBAH_2_PO_4_ consist of only two receptor species, namely the free form and the bound complex, **3b**·H_2_PO_4_^–^. Support for the existence of two components in solution came from a UV-Vis study,^24^ also carried out in CHCl_3_. When the total concentration of **3b** was held constant, isosbestic behaviour was seen upon titration with TBAH_2_PO_4_. This is as expected for a receptor solution consisting of two interconverting species.^24^

Mass spectrometric (MS) analyses provided evidence that receptor **3b** would interact with the H_2_PO_4_^–^ anion in the gas phase. In these studies, an equimolar mixture of **3b** and TBAH_2_PO_4_ in CHCl_3_ was directly injected into the MS instrument without passing the species through a column. Negative ion electrospray ionization (ESI^–^) was used. The high-resolution MS analysis revealed two expected signals, namely, one at *m*/*z* 505.1885 corresponding to [**3b**-H]^–^ and one at *m*/*z* 603.1643 ascribable to the H_2_PO_4_^–^ complex ([**3b**·H_2_PO_4_^–^]). (*cf.* Fig. S4†).

**Fig. 3:**
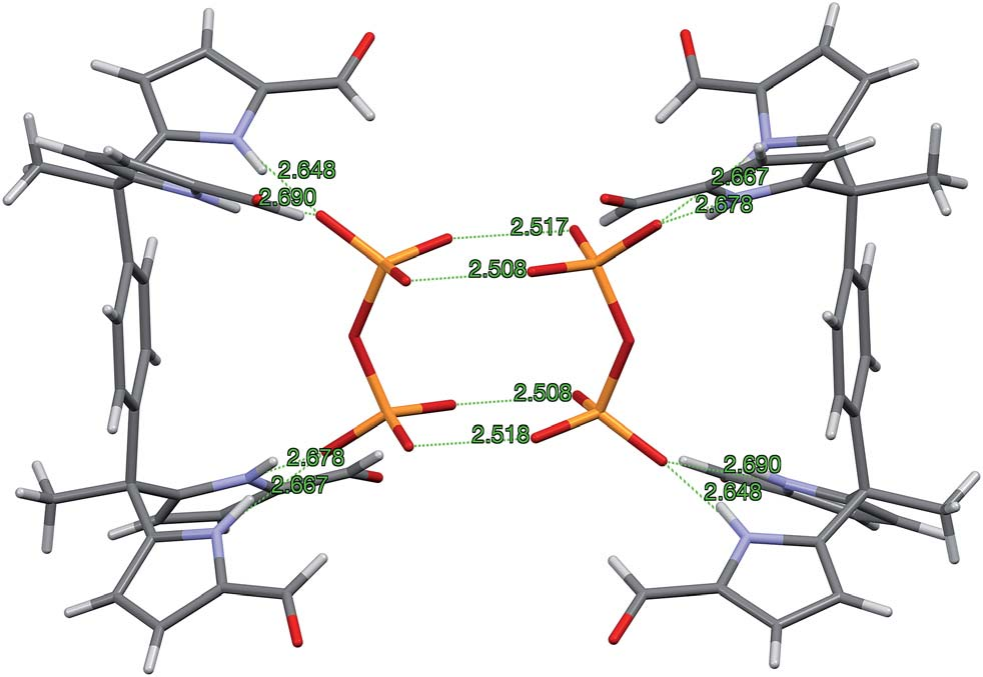
Single crystal structure of the 2 : 2 complex [**3b**·H_2_P_2_O_7_^2–^]_2_ viewed from the side relative to the bridging benzene rings. Two TBA^+^ counter cations and the disordered CH_2_Cl_2_ solvent molecules per pyrophosphate are omitted for clarity.

Further insights into the binding modes operative in the case of the present series of receptors came from single crystal X-ray diffraction analyses.^26^ Diffraction-grade single crystals of the pyrophosphate complex, **3b**·(TBA)_2_H_2_P_2_O_7_, were obtained by layering a solution of **3b**·H_2_P_2_O_7_^2–^ in CH_2_Cl_2_ with *n*-pentane. The complex **3b**·H_2_P_2_O_7_^2–^ in CH_2_Cl_2_ was prepared by treating **3b** with (TBA)_3_HP_2_O_7_. This served to improve the net solubility by converting the poorly soluble receptor (*vide supra*) to the corresponding pyrophosphate complex. In the solid state, a 2 : 2 complex, [**3b**·H_2_P_2_O_7_^2–^]_2_ is seen. The overall complex consists of a hydrogen-bonded dimer that lies across a crystallographic inversion centre (*cf.*Fig. 3).

The protonation state of the bound pyrophosphate in the complex [**3b**·H_2_P_2_O_7_^2–^]_2_ was deduced from the presence of two TBA^+^ counter cations per pyrophosphate dianion. The H_2_P_2_O_7_^2–^ anion lies parallel to the π-surface of the bridging benzene ring. Both diformyl-DPM arms are oriented such that four hydrogen bonds between the diformyl-DPM units in **3b** and the bound H_2_P_2_O_7_^2–^ are favoured. The N–H···O distances between the NH protons of **3b** and the O atoms of H_2_P_2_O_7_^2–^ range from 2.648 to 2.690 Å, separations that are short compared to typical N–H···O distances.^27^,^28^ Each bound H_2_P_2_O_7_^2–^ interacts with another pyrophosphate anion with the result that a hydrogen bonded dimer is formed. In the dimer, four protons are shared between eight oxygen atoms. The observed O···O distances of 2.508–2.518 Å are within the range expected for this type of proton bridged O–H···O type interaction.^28^–^30^ Because the hydrogen atoms on H_2_P_2_O_7_^2–^ are involved in dimer formation, the formyl groups do not participate in the hydrogen bonds that serve to bind the anion. Indeed, the formyl groups point away from the bound H_2_P_2_O_7_^2–^ guest.

**Fig. 4:**
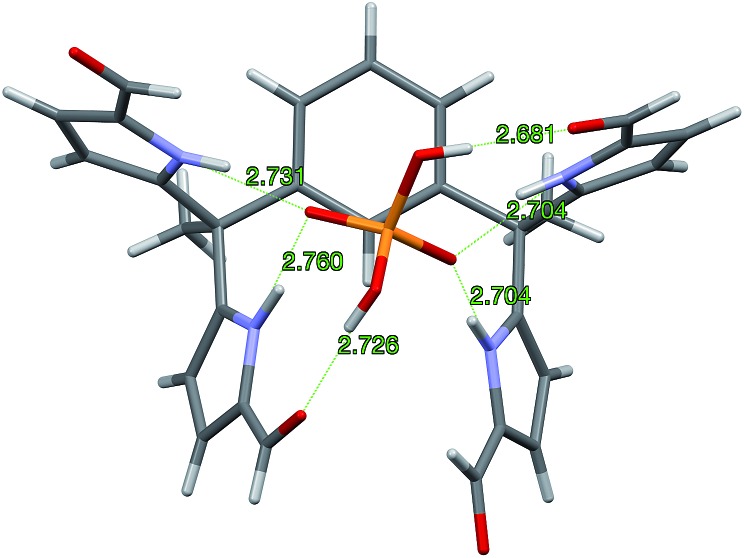
Single crystal structure of the 1 : 1 complex **4b**·H_2_PO_4_^–^ viewed from the top of the bound H_2_PO_4_^–^ molecular ion. One TBA^+^ counter cation is associated with each complex. It and a CHCl_3_ solvent molecule are omitted for clarity.

Diffraction grade single crystals of the complex **4b**·TBAH_2_PO_4_ were obtained in a similar way. The resulting structure is shown in Fig. 4. There is no evidence of dimerization in this case. The lack of dimerization is ascribed to the relatively tight binding between the TBA^+^ counter cation and the bound dihydrogenphosphate guest, which precludes further inter-complex interactions. In the solid state, complex **4b**·H_2_PO_4_^–^ displays the same conformation for the diformyl-DPM units as observed in **3b**·H_2_P_2_O_7_^2–^. The four N–H···O distances that characterize the interaction between the NH protons of **4b** and the O atoms of H_2_PO_4_^–^ range from 2.704 to 2.760 Å. The two O–H···O distances associated with the hydrogen bonding between the formyl groups and the hydrogen atoms of H_2_PO_4_^–^ are 2.681 and 2.726 Å, respectively. Overall, derivative **4b** stabilizes six hydrogen bonding interactions with the H_2_PO_4_^–^ anion. Specifically, it acts as a four-fold donor *via* the pyrrolic NH protons and acts as a two-fold acceptor *via* the two formyl moieties.

**Fig. 5:**
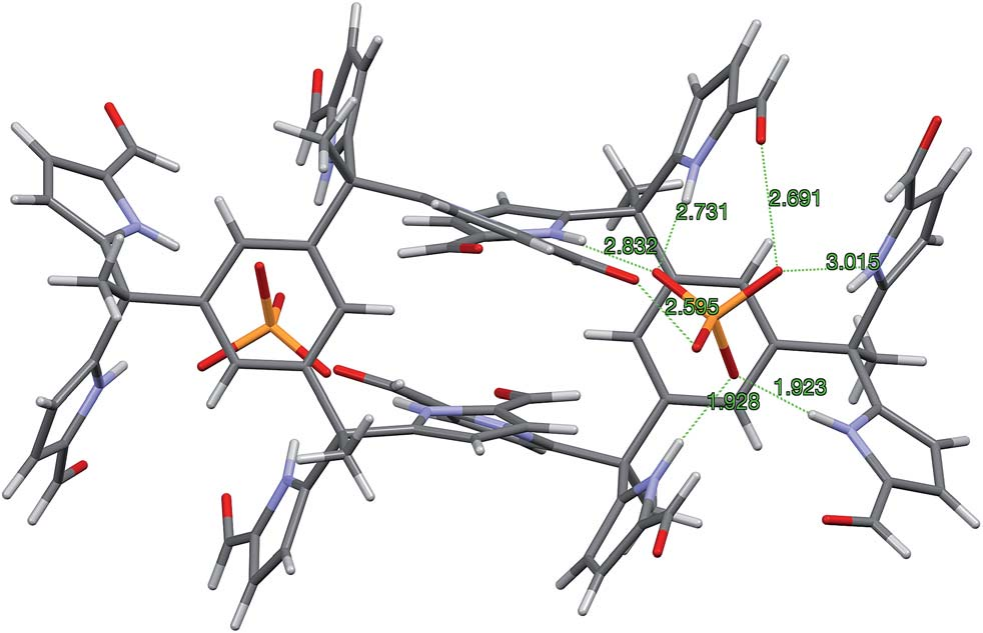
Single crystal structure of the 2 : 2 complex [**7b**·H_2_PO_4_^–^]_2_. One TBA^+^ counter cation per H_2_PO_4_^–^ and 1.5 molecules of CHCl_3_ per **7b** have been omitted for clarity.

Single crystals of complex **7b**·H_2_PO_4_^–^ were obtained by using TBAH_2_PO_4_ to solubilize receptor **7b** in CHCl_3_ (by conversion to the corresponding dihydrogenphosphate complex) and then layering with *n*-pentane. The resulting structure is shown in Fig. 5. Although some analogy to that of **4b**·H_2_PO_4_^–^ discussed above is seen as regard the diformyl DPM-dihydrogenphosphate interactions, in **7b**·H_2_PO_4_^–^, the third diformyl-DPM of **7b** is too distant to interact well with the bound H_2_PO_4_^–^ anion. It thus interacts in an intermolecular sense with a dihydrogenphosphate anion bound to a second receptor. The result is a dimeric complex with overall 2 : 2 stoichiometry. The four pyrrole NH-to-deprotonated phosphate oxygen N–H···O distances range from 2.730 to 2.827 Å. The intermolecular hydrogen bond that is presumed to play a role in stabilizing the 2 : 2 complex is characterized by an O–H···O distance of 2.598 Å.

**Fig. 6:**
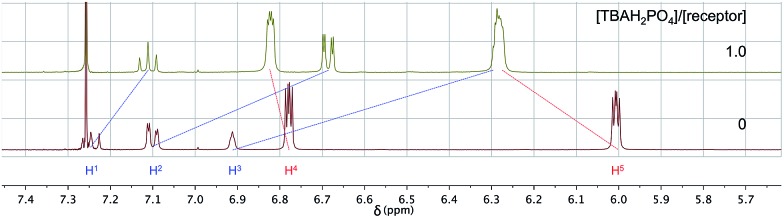
Partial ^1^H NMR spectra of receptor **4b** before (bottom) and after (top) addition of one molar equivalent of TBAH_2_PO_4_ in CDCl_3_. Dashed lines show the changes in the chemical shifts of selected proton signals ascribable to receptor **4b**.

Based on the binding mode observed in the solid state, we considered it likely that anion–π interactions, as well as pyrrole NH–anion hydrogen bonding interactions contribute to anion binding. To explore this possibility, the effect of adding TBAH_2_PO_4_ to the most soluble receptor of the present series, namely the *meta*-derivative **4b**, was investigated by ^1^H NMR spectroscopy (Fig. 6). Upon addition of one molar equivalent of TBAH_2_PO_4_ to a CDCl_3_ solution of **4b**, discernible shifts in the proton resonances of **4b** were observed (*cf.*Table 2). Of particular note were the 0.6 ppm upfield shifts seen for the proton signals of the linking benzene ring. Similar ^1^H NMR spectroscopic studies of **5b** carried out in CDCl_3_, **7b** in a more competitive solvent mixture, CDCl_3_ : DMSO-*d*_6_ (8 : 1), likewise revealed upfield shifts in the intervening benzene resonances (*cf.* Fig. S8 and S10†). Such shifts are consistent with the decrease in the deshielding effect of the intervening aromatic ring current as seen in previous studies of α,α,α,α-*meso*-tetraaryl-C[4]P derivatives by Ballester and co-workers.^31^ However, as noted by a reviewer, such an observation is not sufficient to confirm or rule out the presence of an anion–π interaction. Intuitively, the interaction of anion H_2_PO_4_^–^ with the electron-rich intervening benzene ring of **4b** is expected to be repulsive, thus destabilizing the overall binding event. However, Deyà^32^ has suggested that the polarization of a π-electron system by an anion induces a dipole moment that may provide a contribution to the anion–receptor interaction. In the case of the present system, further studies will be necessary to determine whether anion–π interactions (if any) are playing a substantial role in mediating the observed anion recognition behaviour.

**Table 2:** Summary of chemical shifts of selected proton resonances of **4b** before and after addition of one molar equivalent of TBAH_2_PO_4_ in CDCl_3_

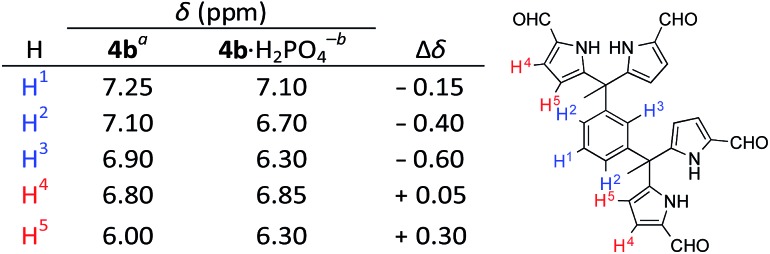

^*a*^The solution of **4b** was prepared from diffraction grade single crystals of **4b**, which contained two DMF molecules of crystallization per equivalent of receptor.

^*b*^Obtained by treating with the TBA^+^ salt of H_2_PO_4_^–^; see text for details.

Receptor **7b** proved even less soluble in neat, non-hydrogen bonding solvents, such as CHCl_3_, than its congeners **3b–5b**. This made it impossible to carry out binding studies that were directly comparable to those reported earlier.^24^ However, in analogy to what was seen in the case of these other receptors, it was found that the solubility of **7b** increased upon the addition of TBAH_2_PO_4_. Dilute solutions of **7b**·H_2_PO_4_^–^ in CHCl_3_ could thus be prepared by mixing single crystals of **7b** with *ca.* 2.75 molar equivalents of TBAH_2_PO_4_. The UV-Vis spectrum of this solution was characterized by features similar to those seen in the reported spectrum of **3b**·H_2_PO_4_^–^ at analogous concentrations. Thus, the observed spectral features were ascribed to the complex **7b**·H_2_PO_4_^–^. When the CHCl_3_ solution made up from **7b** and TBAH_2_PO_4_ was titrated with CH_3_OH, spectral changes and isosbestic behaviour were observed. On this basis, we conclude that the H_2_PO_4_^–^ affinity of **7b** decreases in the presence of increasing quantities of CH_3_OH (Fig. S15†). However, it was also apparent that large quantities of methanol were required to disrupt the complex completely. It was thus decided that a solution of 3% (v/v) CH_3_OH in CHCl_3_ would provide a good balance between solubility and binding affinity and allow for quantitative analyses.

**Fig. 7:**
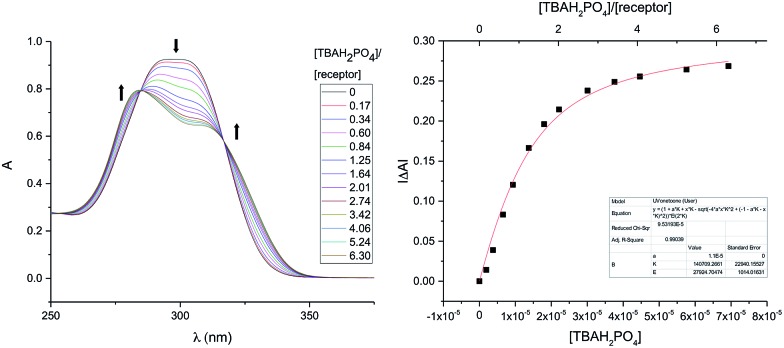
Left: Stacked UV-Vis spectra for titration of **7b** (1.1 × 10^–5^ M) with TBAH_2_PO_4_ (2.9 × 10^–4^ M) in CHCl_3_ containing 3% (v/v) CH_3_OH. Right: Binding curve fitting plotted from titration data at 302 nm. *K*_a_ = (1.4 ± 0.2) × 10^5^ M^–1^.

In a first set of experiments, an 11 μM solution of **7b** in 3% (v/v) CH_3_OH in CHCl_3_ was prepared and then subjected to titration with TBAH_2_PO_4_ (*cf.*Fig. 7). The induced spectral changes were then fit to a 1 : 1 binding profile and used to calculate the binding constant corresponding to H_2_PO_4_^–^ binding in accord with the methods described previously.^24^ Similar UV-Vis titrations were carried out with a series of anions in the form of their respective TBA salts. It was found that titration with either the H_2_PO_4_^–^ or HP_2_O_7_^3–^ anions gave rise to significant spectral changes with saturation behaviour being observed in the presence of fewer than ten molar equivalents of these two anions. On the other hand, no discernable spectral changes were seen upon titration with HSO_4_^–^, Cl^–^, or NO_3_^–^.

In the initial studies of **3b–5b**, CHCl_3_ was used as the solvent. To allow for a comparison between the original *tetrakis*-(1*H*-pyrrole-2-carbaldehyde) anion receptors and the newer and *hexakis* analogue **7b**, compound **3b** was subject to titration with H_2_PO_4_^–^ in 3% (v/v) CH_3_OH–CHCl_3_. The results from this comparison are summarised in Table 3. Briefly, **7b** is characterized by a *ca.* 2 orders of magnitude higher dihydrogenphosphate anion affinity. This increase is in accord with the design expectations underlying **7b**, namely that the presence of a third DPM subunit would force this system to adopt a preorganised conformation in a way that isn't possible for **3b**.

**Table 3:** Summary of calculated binding affinities, *K*_a_ (M^–1^) as determined by UV-Vis spectroscopy in two separate solvent systems^*a*^

Anions^*a*^	**3b** ^*b*^	**3b** ^*c*^	**7b** ^*c*^
H_2_PO_4_^–^	(8 ± 2) × 10^6^	(2.1 ± 0.3) × 10^3^	(1.4 ± 0.2) × 10^5^
HP_2_O_7_^3–^	(1.4 ± 0.1) × 10^5^	(3.8 ± 0.6) × 10^3^	(3.6 ± 0.2) × 10^4^
HSO_4_^–^	(4.1 ± 0.2) × 10^3^	nd	nd
NO_3_^–^	nd	nd	nd
Cl^–^	nd	nd	nd

^*a*^The anions were studied as their TBA^+^ salts.

^*b*^In CHCl_3_; data from ref. 24.

^*c*^In CHCl_3_ containing 3% (v/v) CH_3_OH.

**Fig. 8:**
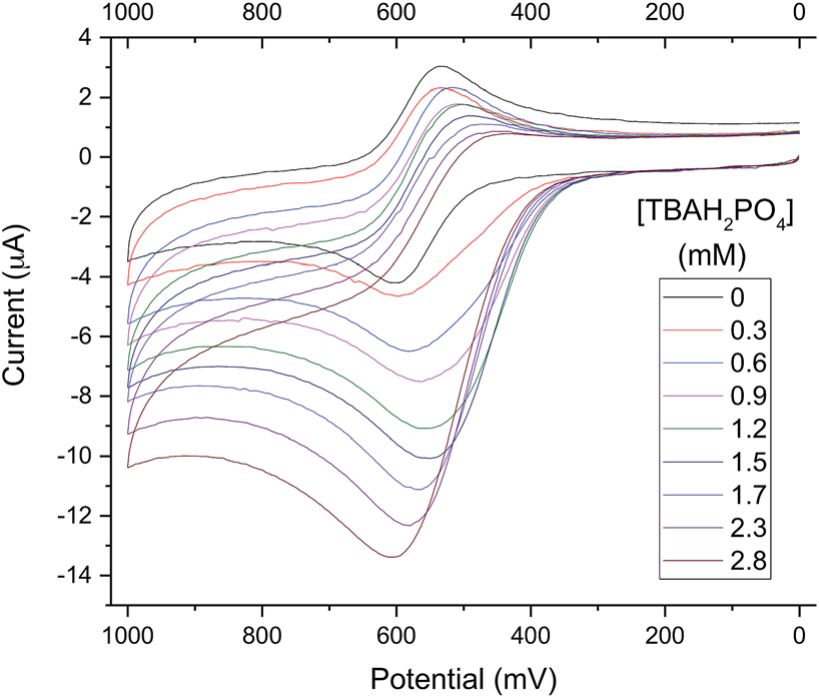
Stacked cyclic voltammogram of **6b** in CH_3_CN containing 10% (v/v) DMF. The initial turbid mixture of **6b** contained a quantity of material sufficient to produce a 1.0 mM solution once completely dissolved. This mixture was titrated with TBAH_2_PO_4_ until the TBAH_2_PO_4_ concentration reached 2.8 mM. TBAPF_6_ (0.1 M) was used as the supporting electrolyte. Glassy carbon was used as the working electrode, a Pt wire as the counter electrode, and a Ag/AgCl couple as the reference electrode.

To exploit the binding characteristics of the present diformyl-DPM receptors for the purposes of anion sensing, analogue **6b** was prepared. It incorporates a ferrocene spacer and was designed to perform as an electro-active analogue of the *tetrakis*(1*H*-pyrrole-2-carbaldehyde) receptors **3b–5b**. This new derivative proved poorly soluble in typical organic solvents used for electrochemical studies (*e.g.*, CH_3_CN, DMF, CH_2_Cl_2_). However, as above, an increase in its solubility was seen upon the addition of TBAH_2_PO_4_. This was seen as a “useful property”^33^ that could be used to obtain insights into the H_2_PO_4_^–^ binding events under conditions of electrochemical analysis. It was found, for instance, that initial turbid mixtures of free receptor **6b** in CH_3_CN containing 10% (v/v) DMF give rise to a quasi-reversible cyclic voltammogram (CV) (black line in Fig. 8). The oxidation wave for **6b** becomes increasingly irreversible as the relative concentration of TBAH_2_PO_4_ increases. Eventually, the CV becomes characterized by a relatively intense anodic peak and a near-absence of a corresponding cathodic feature.

The observed increase in the anodic peak current is ascribed to the increase in the concentration of **6b** (both free and complexed) that occurs as the titration runs its course. Since the **6b** is not completely soluble in the solvent mixture in the absence of H_2_PO_4_^–^, the concentration of free **6b** in the titration mixture should be constant up until the point where all traces of solid **6b** are dissolved, presumably by conversion to the corresponding dihydrogenphosphate complex, **6b**·H_2_PO_4_^–^. To the extent such a supposition is correct, it would account for the observed increase in the peak current, which could be attributed directly to the formation of the readily oxidizable species, **6b**·H_2_PO_4_^–^. When the increase in the anodic peak current is plotted against the added H_2_PO_4_^–^ concentration, a binding isotherm could be obtained (*cf.* Fig. S20†) from which an approximate H_2_PO_4_^–^ affinity, (1.0 ± 0.1) × 10^3^ M^–1^, could be calculated (*cf.* Table ESI-1†). The loss in reduction signal intensity is believed to reflect the strong interaction between the oxidized ferrocenium receptor (**6b**^+^) and H_2_PO_4_^–^.

## Conclusions

**Fig. 9:**
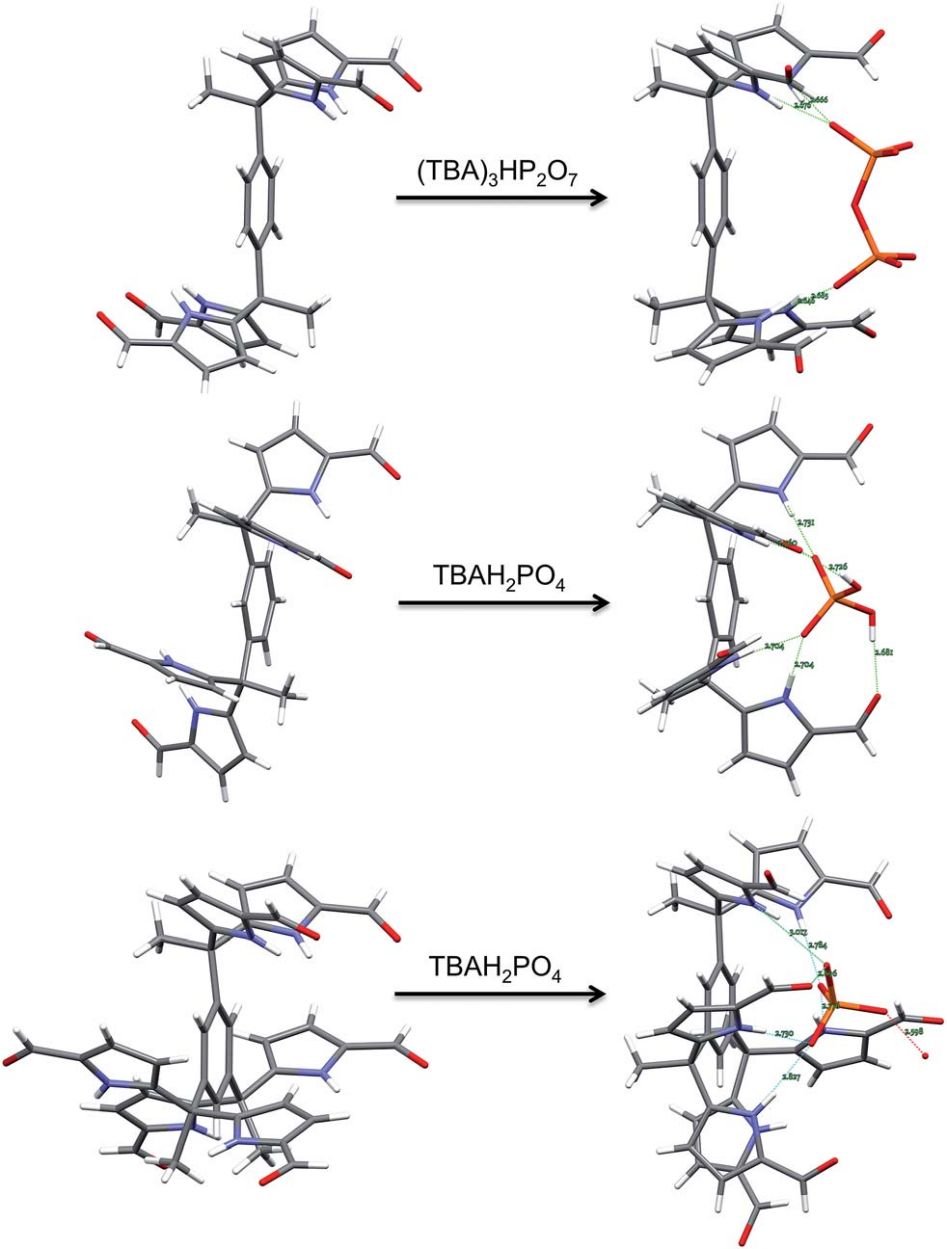
Views of the single crystal structures of **3b**, **4b**, and **7b** in the absence and presence of bound phosphate derivatives.

In summary, we have extended the *tetrakis*(1*H*-pyrrole-2-carbaldehyde) receptor family to include a new electroactive derivative, **6b**, incorporating a ferrocene linker. Also reported here is a hexaformyl trisDPM derivative, **7b**. The anion binding properties of these new systems were studied by monitoring the increase in solubility electrochemically, through high-resolution mass spectrometric analyses, 1D ^1^H NMR and 2D DOSY spectroscopic experiments, single crystal X-ray diffraction analyses, and UV-Vis spectroscopic titrations. Conformational switching from *trans*-like conformations as seen in the free *tetrakis*- (**3b**,**4b**) and *hexakis-* (**7b**) (1*H*-pyrrole-2-carbaldehyde) receptors to the corresponding *cis*-like conformations upon interacting with phosphate derivatives was revealed by X-ray diffraction analyses in the solid state (Fig. 9). In mixed methanol–chloroform solution, the new *hexakis*-carbaldehyde derivative **7b** was found to exhibit an affinity for H_2_PO_4_^–^ that is roughly 100× higher than that displayed by the *tetrakis* analogue **3b**. This increase in binding affinity is rationalized in terms of a receptor design that overcomes in part the conformational penalty that needs to be paid in order to achieve substrate binding. As such, the present findings provide experimental insights that might prove useful in the design of anion receptors based on rather simple binding motifs, such as pyrroles and dipyrromethane subunits.

## Supplementary Material

Supplementary information

Crystal structure data
