# Supplementary material for: Non-cyclic formylated dipyrromethanes as phosphate anion receptors
Source: Chem Sci. 2016 Feb 24;7(6):3843–50. doi: 10.1039/c6sc00015k (PMC6013804; doi:10.1039/c6sc00015k)
Supplement: Supplementary file 1 [file SC-007-C6SC00015K-s001.pdf]

Electronic Supplementary Material (ESI) for Chemical Science.
This journal is © The Royal Society of Chemistry 2016

# Electronic Supporting Information for

# Non-cyclic Formylated Dipyrromethanes as Phosphate Anion Receptors

Murat K. Deliomeroglu,[a] Vincent M. Lynch,[a] and Jonathan L. Sessler*[a]

## Table of Contents

General .......... 2
Syntheses of Compounds 6a–7b .......... 2
Single Crystal X-ray Diffraction Analyses .......... 5
Anion Binding Studies .......... 7
Mass Spectrometry .......... 7
$^{1}$H NMR Spectroscopy .......... 10
UV-Vis Spectroscopy .......... 13
Cyclic Voltammetry .......... 22
X-ray Diffraction Analyses .......... 27
Experimental Details for X-ray Diffraction Analysis .......... 27
Structure Refinement Parameters .......... 36
$^{1}$H and $^{13}$C NMR Spectra .......... 41
Notes and References .......... 46

[a] M. K. Deliomeroglu, Dr. V. M. Lynch, Dr. J. L. Sessler
Department of Chemistry, The University of Texas at Austin
105 E. 24th Street-Stop A5300, Austin, Texas 78712-1224 (USA)
E-mail: Sessler@cm.utexas.edu

## General

1,1'-Diacetylferrocene was obtained from Ark Pharm, Inc. All other solvents and chemicals were purchased from Aldrich, TCI, and Acros. Pyrrole was distilled at 80 °C under reduced pressure (100 mbar) prior to use. Other solvents and reagents were used without further purification. Compounds **3b** and **4b** were prepared as reported previously.[1] The NMR spectra were referenced to residual protic solvent peaks. All NMR spectroscopic solvents were purchased from Cambridge Isotope Laboratories.

## Syntheses of Compounds 6a–7b

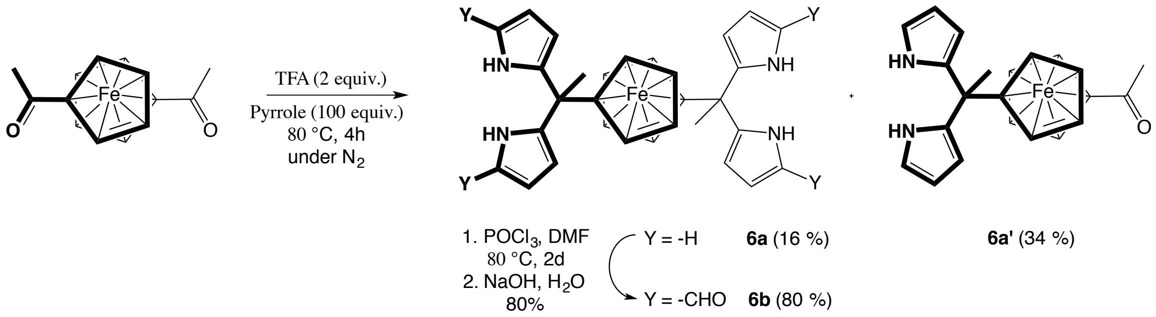


1,1'-Bis(1,1-di(1*H*-pyrrol-2-yl)ethyl)ferrocene (**6a**):

1,1'-Diacetylferrocene (500 mg, 1.85 mmol) was dissolved in pyrrole (12 mL, 185 mmol). Dry nitrogen gas was bubbled through this solution for 10 min. Trifluoroacetic acid (TFA) (0.3 mL, 3.9 mmol) was added to the mixture and the mixture was heated to 80°C for 4 hours while bubbling with $N_2$. Note: Proper ventilation is required since TFA is a corrosive and low boiling substance. The reaction mixture was then cooled to room temperature and dissolved in dichloromethane (DCM). The organic solution was extracted with 0.5 M NaOH to remove the TFA. The organic layer was dried over anhydrous $MgSO_4$ and the solvent was removed. Unreacted pyrrole was recycled from this crude product mixture by distillation at 80 °C and 100 mbar. Column chromatography (silica gel) was performed using 15:1 DCM:EA (ethyl acetate) as the eluent. The yellow

fraction (Rf = 0.95) was collected and gave a yellowish green oil upon evaporation (150 mg, 16%). The oil was solidified in 1 ml of methanol. $^{1}H$ NMR (400 MHz, $CDCl_3$) δ 7.78 (bs, 4H), 6.59 (m, 4H), 6.11 (m, 4H), 5.93 (m, 4H), 4.08 (t, $J$ = 1.9 Hz, 4H), 3.96 (t, $J$ = 1.9 Hz, 4H), 1.95 (s, 6H) ppm. $^{13}C$ NMR (100 MHz, $CDCl_3$) δ 138.32, 116.51, 107.97, 105.48, 98.08, 68.97, 67.90, 39.48, 28.64 ppm. HRMS ($CI^+$) *m/z* for $C_{30}H_{30}FeN_4$ $[M]^+$ calcd 502.1820, found 502.1826.

1-Acetyl-1'-(1,1-di(1H-pyrrol-2-yl)ethyl)ferrocene (**6a'**):

This product was obtained as a side product from the above reaction mixture. The product is a red solid (240 mg, 34%) that elutes from the column after **6a** (for **6a'** Rf = 0.5). $^{1}H$ NMR (400 MHz, $CDCl_3$) δ 7.99 (bs, 2H), 6.66 (m, 2H), 6.12 (m, 2H), 5.91 (m, 2H), 4.73 (t, $J$ = 2.0 Hz, 2H), 4.46 (t, $J$ = 2.0 Hz, 2H), 4.22 (t, $J$ = 1.9 Hz, 2H), 4.00 (t, $J$ = 1.9 Hz, 2H), 2.31 (s, 3H), 2.00 (s, 3H) ppm. $^{13}C$ NMR (100 MHz, $CDCl_3$) δ 203.05, 137.60, 117.02, 107.87, 105.76, 100.32, 79.47, 75.53, 70.62, 69.53, 68.86, 39.40,29.00, 27.62 ppm. HR-MS-$CI^+$ *m/z* for $C_{22}H_{22}FeN_2O$ $[M]^+$ calcd 386.1082, found 386.1072.

5,5',5'',5'''-(1,1'-Ferrocenylenebis(ethane-1,1,1-triyl))tetrakis(1*H*-pyrrole-2-carbaldehyde) (**6b**):

A Vilsmeier–Haack formylation procedure identical to that reported previously[1] was used, albeit starting with **6a**. The product, **6b**, was obtained in 80% yield. $^{1}H$ NMR (400 MHz, DMSO-$d_6$) δ 11.63 (bs, 4H), 9.41 (s, 4H), 6.83 (m, 4H), 5.67 (m, 4H), 4.07 (m, 4H), 3.87 (m, 4H), 2.02 (s, 6H) ppm. $^{13}C$ NMR (100 MHz, DMSO-$d_6$) δ 178.7, 146.9, 132.3, 109.7, 95.7, 68.1, 67.9, 55.3, 25.0 ppm. $^{13}C$ NMR (100 MHz, DMF-$d_7$) δ 179.3, 147.7, 133.6, 110.5, 96.8, 69.1, 68.9, 41.0, 26.0 ppm. HRMS ($ESI^+$) *m/z* for $C_{34}H_{30}FeN_4O_4$ $[M+H]^+$ calcd 615.16900, found 615.16930.

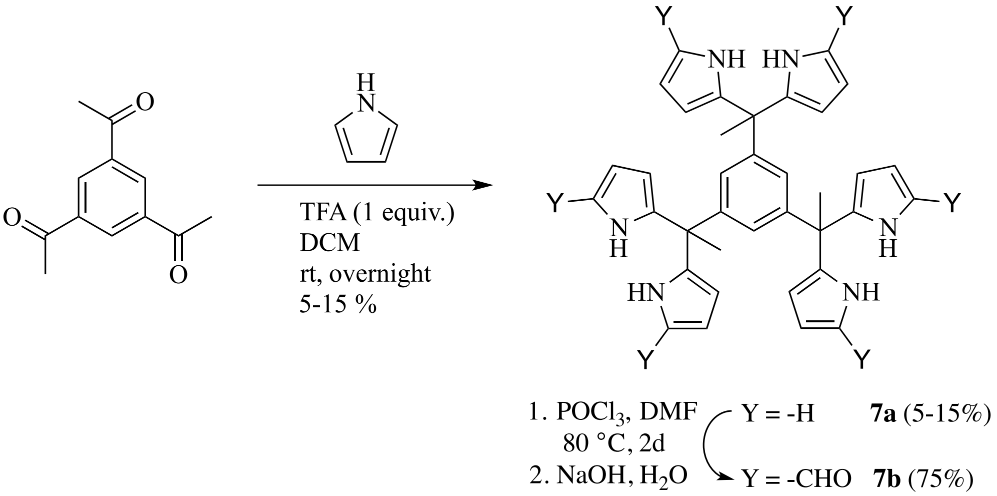


1,3,5-Tris(1,1-di(1*H*-pyrrol-2-yl)ethyl)benzene (**7a**):

A modification of the published dipyrromethane-forming procedure[1] was employed starting with 1,3,5-triacetylbenene and using $CH_2Cl_2$ as a co-solvent. The reaction was carried out at room temperature. A co-solvent is used to reduce the formation of insoluble side products. The desired product **7a** was obtained as colourless oil in 15% yield. $^1$H NMR (400 MHz, $CDCl_3$) δ 7.65 (bs, 6H), 6.90 (s, 3H), 6.54 (m, 6H), 6.13 (m, 6H), 5.89 (m, 6H), 1.95 (s, 9H) ppm. $^{13}$C NMR (100 MHz, $CDCl_3$) δ 146.5, 137.4, 124.8, 117.1, 108.0, 106.3, 44.7, 28.5 ppm. HRMS (ESI$^+$) *m/z* for $C_{36}H_{36}N_6$ $[M+H]^+$ calcd 553.30740, found 553.30730.

5,5',5'',5''',5'''',5'''''-(Benzene-1,3,5-triyltris(ethane-1,1,1-triyl))hexakis(1*H*-pyrrole-2-carbaldehyde) (**7b**):

The Vilsmeier–Haack formylation procedure reported previously was used,[1] albeit starting with **7a**. Product **7b** was obtained in 80% yield. $^1$H NMR (400 MHz, DMSO-$d_6$) δ 11.68 (s, 6H), 9.34 (s, 6H), 6,75 (m, 6H), 6.42 (s, 3H), 5.74 (m, 6H), 1.91 (s, 9H) ppm. $^{13}$C NMR (100 MHz, DMSO-$d_6$) δ 178.87, 145.67, 145.26, 133.03, 124.63, 110.43, 109.59, 45.38, 26.95 ppm. HRMS (ESI$^+$) *m/z* for $C_{42}H_{36}N_6O_6$ $[M+Na]^+$ calcd 743.25890, found 743.25920.

## Single Crystal X-ray Diffraction Analyses

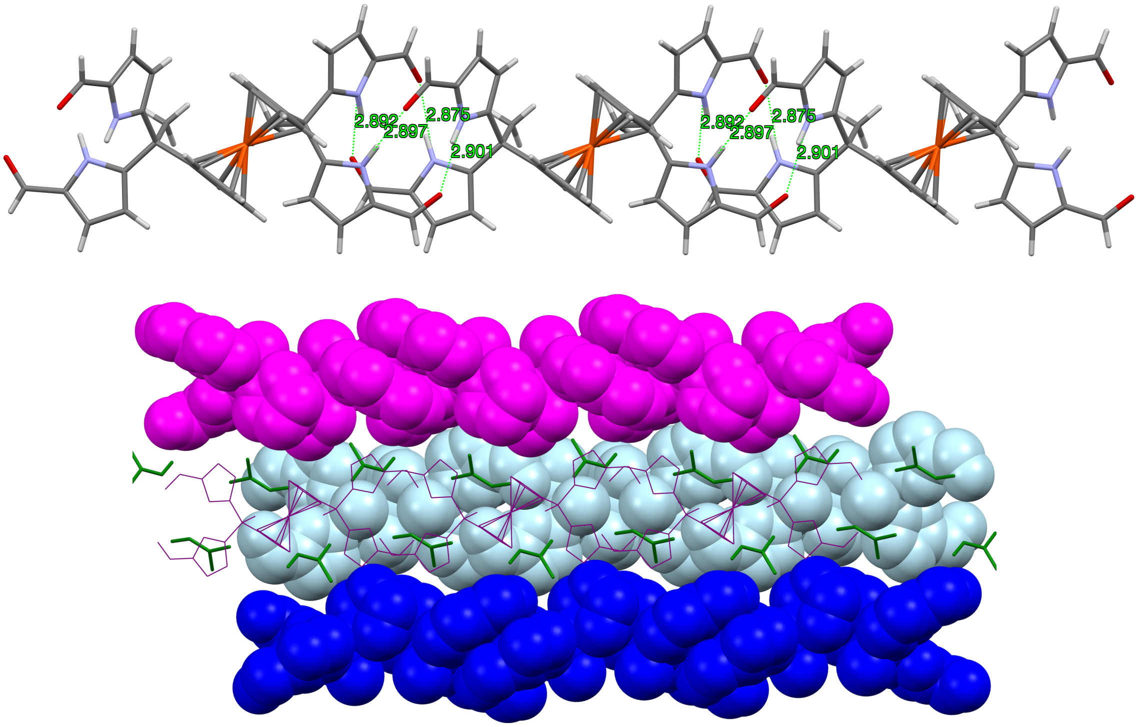


**Fig. SI-1** Top: Linear assembly formed by one of the atropisomers of **6b**. The N-H•••O hydrogen bonding distances range from 2.875 to 2.901 Å. Bottom: Packing of the linear ensembles shown in the top portion of the figure.

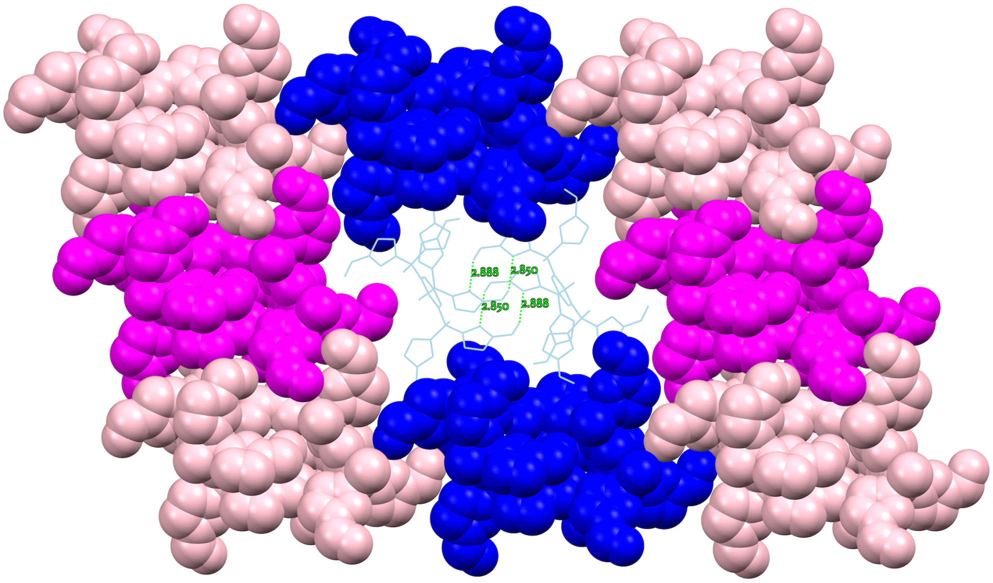


**Fig. SI-2** Hydrogen bond linked dimers of **7b** and their further self-assembly into layers. Each colour indicates one dimer made up from **7b**. The N-H•••O distances for the dimer are 2.850 and 2.888 Å. The dimer in the centre is drawn in wireframe representation. The space between the layers is filled with $CHCl_3$.

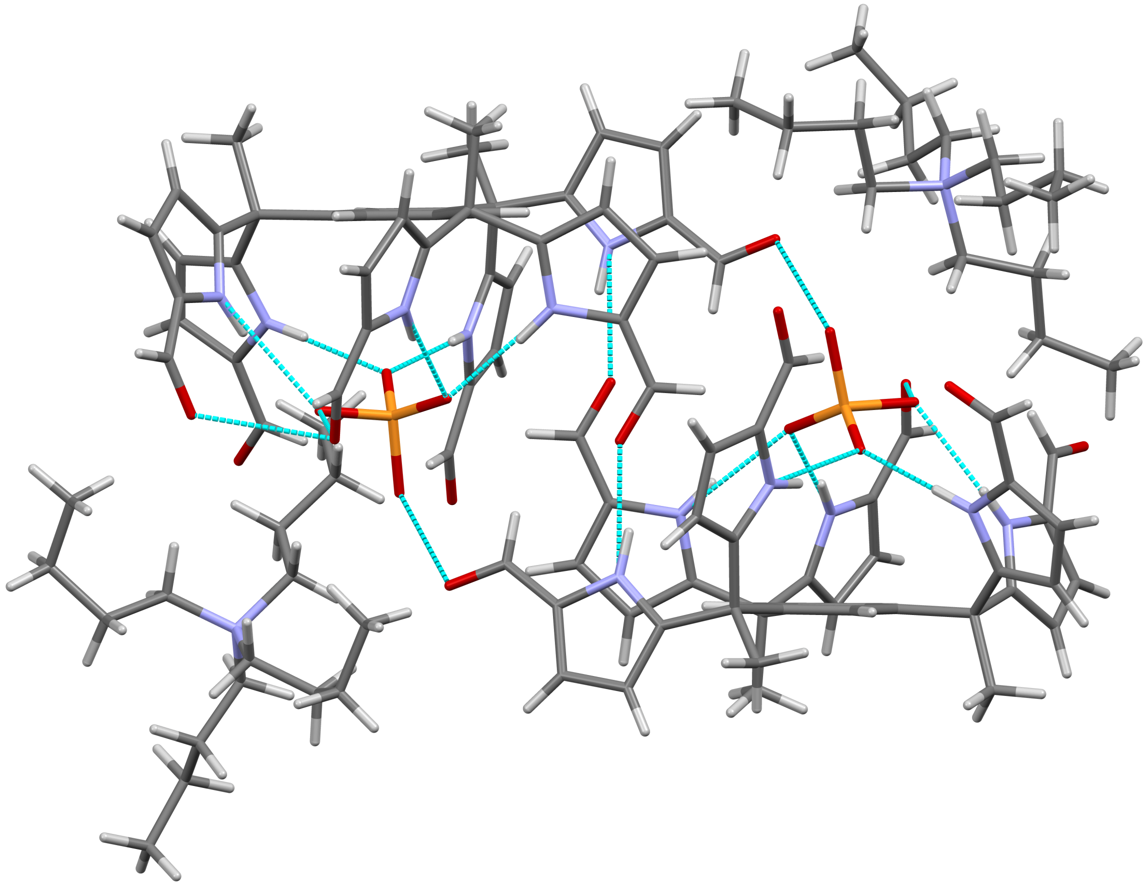

**Fig. SI-3** A different view of complex $(TBA)_2[\mathbf{7b}\cdot H_2PO_4^-]_2$.

## Anion Binding Studies

### Mass Spectrometry

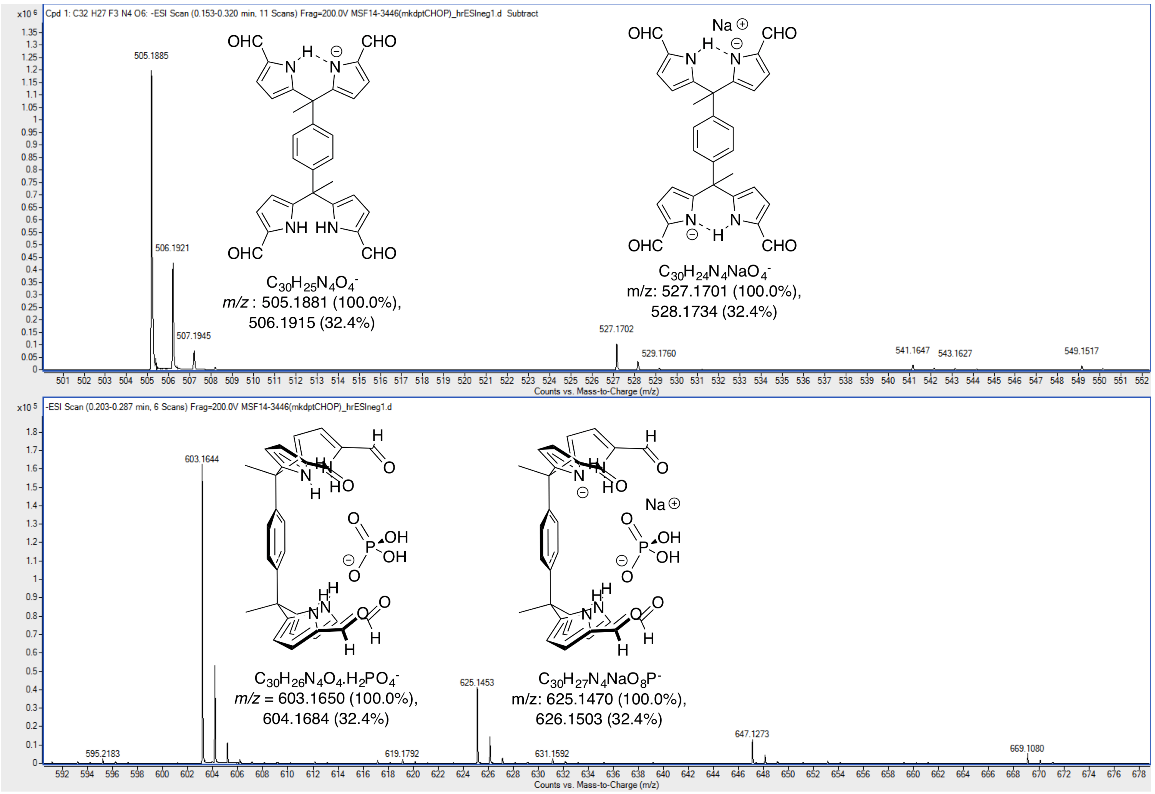


**Fig. SI-4** High-resolution mass spectrum (HR-ESI$^-$-MS) (electrospray ionization in negative mode) of an equimolar mixture of **3b** and $TBAH_2PO_4$ in $CHCl_3$. Note that the top and bottom panels cover different mass ranges.

MS Zoomed Spectrum

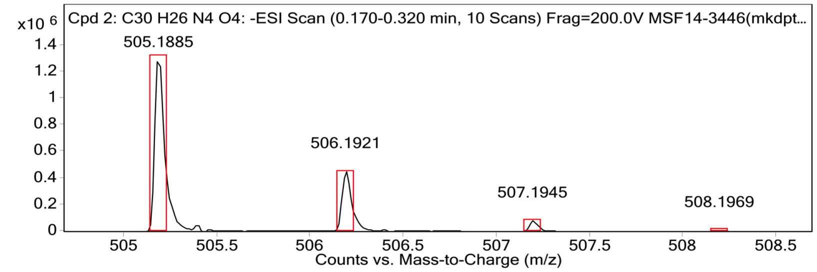


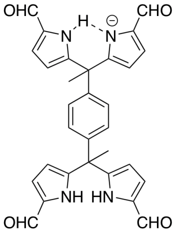


$C_{30}H_{25}N_4O_4^-$
*m/z* : 505.1881 (100.0%), 506.1915 (32.4%)

**MS Spectrum Peak List**

| *Obs. m/z* | Calc. m/z | Charge | Abund | Formula | Ion/Isotope | Tgt Mass Error (ppm) |
|---|---|---|---|---|---|---|
| 505.18850 | 505.18810 | 1 | 1316842.41 | C30H26N4O4 | (M-H)- | -0.66 |
| 506.19210 | 506.19120 | 1 | 453129.97 | C30H26N4O4 | (M-H)- | -1.68 |
| 507.19450 | 507.19410 | 1 | 81999.79 | C30H26N4O4 | (M-H)- | -0.74 |
| 508.19690 | 508.19680 | 1 | 10505.41 | C30H26N4O4 | (M-H)- | -0.17 |
| 509.19830 | 509.19950 | 1 | 1024.64 | C30H26N4O4 | (M-H)- | 2.29 |

--- End Of Report ---

MS Zoomed Spectrum

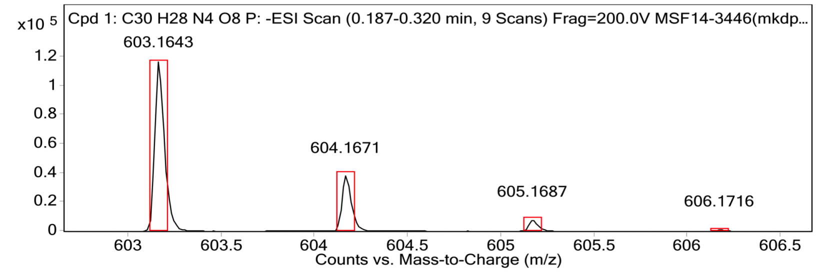


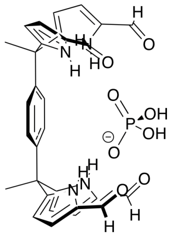


$C_{30}H_{26}N_4O_4.H_2PO_4^-$
*m/z* = 603.1650 (100.0%), 604.1684 (32.4%)

**MS Spectrum Peak List**

| *Obs. m/z* | Calc. m/z | Charge | Abund | Formula | Ion/Isotope | Tgt Mass Error (ppm) |
|---|---|---|---|---|---|---|
| 505.18840 | | | 1447258.67 | | | |
| 603.16430 | 603.16500 | 1 | 117411.72 | C30H28N4O8P | M- | 1.15 |
| 604.16710 | 604.16810 | 1 | 38138.4 | C30H28N4O8P | M- | 1.77 |
| 605.16870 | 605.17080 | 1 | 8072.14 | C30H28N4O8P | M- | 3.57 |
| 606.17160 | 606.17340 | 1 | 1396.14 | C30H28N4O8P | M- | 3.02 |

--- End Of Report ---

**Fig. SI-5** HR-ESI$^-$-MS reports for [**3b**-H]$^-$ and [**3b**$\cdot H_2PO_4^-$].

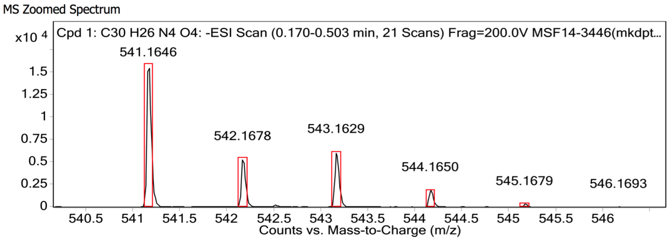


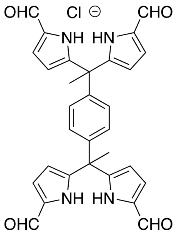


**MS Spectrum Peak List**

| Obs. m/z | Calc. m/z | Charge | Abund | Formula | Ion/Isotope | Tgt Mass Error (ppm) |
|---|---|---|---|---|---|---|
| 505.18850 | | | 764435.43 | | | |
| 541.16460 | 541.16480 | 1 | 15949.35 | C30H26N4O4 | (M+Cl)- | 0.42 |
| 542.16780 | 542.16790 | 1 | 5386.73 | C30H26N4O4 | (M+Cl)- | 0.14 |
| 543.16290 | 543.16340 | 1 | 6184.62 | C30H26N4O4 | (M+Cl)- | 0.92 |
| 544.16500 | 544.16560 | 1 | 1921.94 | C30H26N4O4 | (M+Cl)- | 1.19 |
| 545.16790 | 545.16820 | 1 | 358.74 | C30H26N4O4 | (M+Cl)- | 0.6 |
| 546.16930 | 546.17080 | 1 | 48.54 | C30H26N4O4 | (M+Cl)- | 2.76 |

--- End Of Report ---

$C_{30}H_{26}ClN_4O_4^-$
*m/z* : 541.1648 (100.0%), 542.1682 (32.4%), 543.1619 (32.0%), 544.1652 (10.4%)

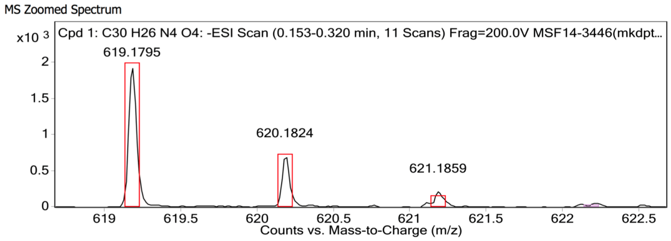


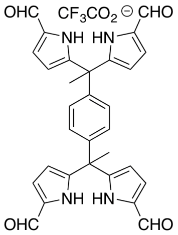


**MS Spectrum Peak List**

| Obs. m/z | Calc. m/z | Charge | Abund | Formula | Ion/Isotope | Tgt Mass Error (ppm) |
|---|---|---|---|---|---|---|
| 505.18850 | | | 1234253.24 | | | |
| 619.17950 | 619.18100 | 1 | 1950.34 | C30H26N4O4 | (M+CF3COO)- | 2.48 |
| 620.18240 | 620.18410 | 1 | 709.89 | C30H26N4O4 | (M+CF3COO)- | 2.82 |
| 621.18590 | 621.18690 | 1 | 219.56 | C30H26N4O4 | (M+CF3COO)- | 1.71 |

--- End Of Report ---

$C_{32}H_{26}F_3N_4O_6^-$
*m/z* : 619.1810 (100.0%), 620.1843 (34.6%)

**Fig. SI-6** HR-ESI$^-$-MS reports for two observed minor adducts, [**3b**·$Cl^-$] and [**3b**·$CF_3CO_2^-$], that are thought to arise as the result of residual contaminants present in the MS apparatus.

## $^1$H NMR Spectroscopy

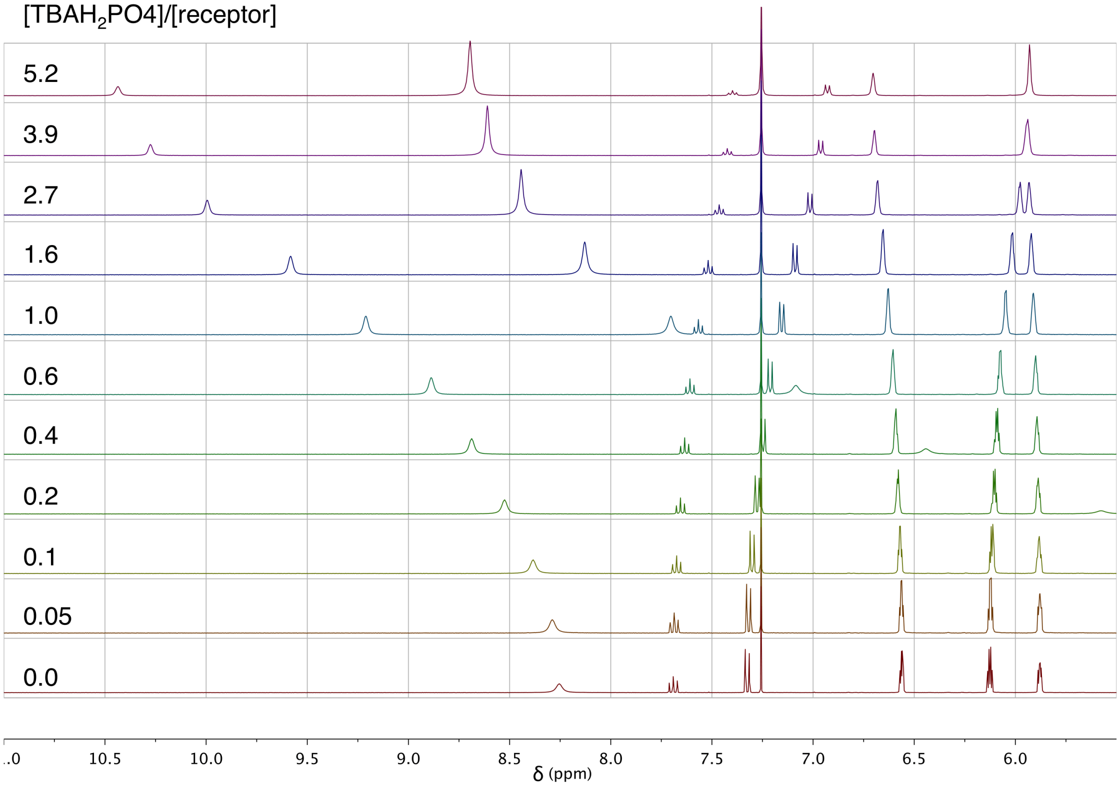


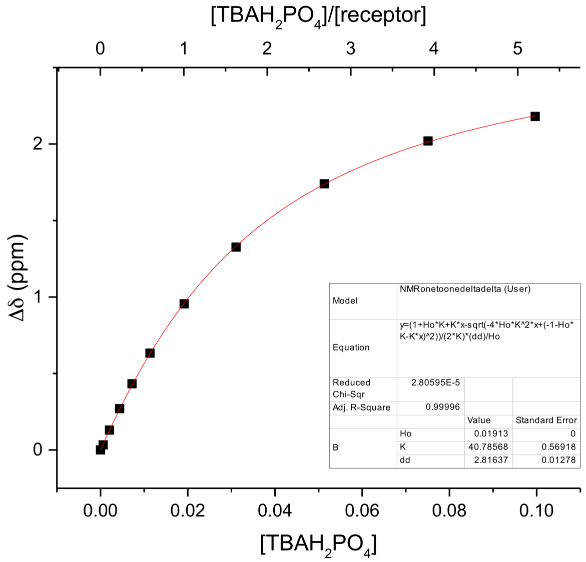


**Fig. SI-7** Top: Stacked $^1$H NMR spectra corresponding to the titration of **5a** (19.1 mM) with $TBAH_2PO_4$ (0.235 M) in $CDCl_3$. Bottom: Binding curve obtained by fitting the chemical shift change of the pyrrole NH signal against [$TBAH_2PO_4$]. $K_a = 40.8 \pm 0.6\ M^{-1}$.

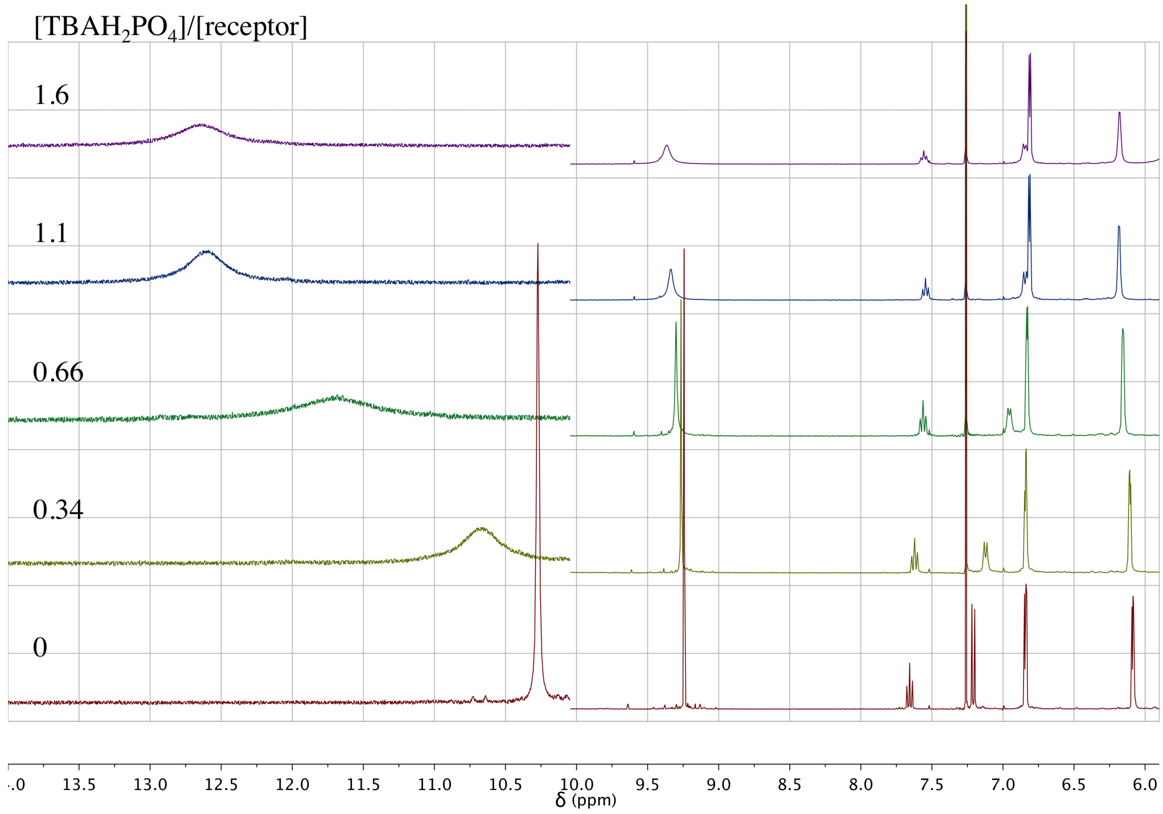


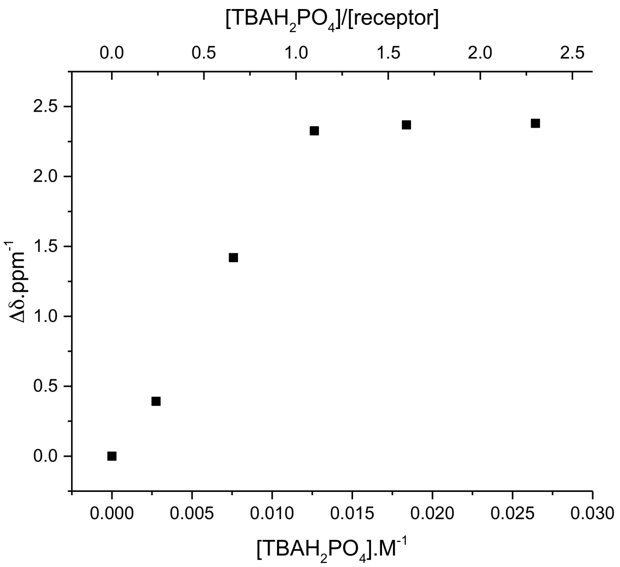


**Fig. SI-8** Top: Stacked $^{1}$H NMR spectra of receptor **5b** (11.5 mM) with varying quantities of $H_2PO_4^-$ recorded in $CDCl_3$. The doublet arising from aromatic hydrogen atoms of the benzene ring of receptor **5b** displays the largest upfield shift (Δδ = -0.36 ppm). Bottom: The chemical shift change of the pyrrole NH signal plotted against [$TBAH_2PO_4$].

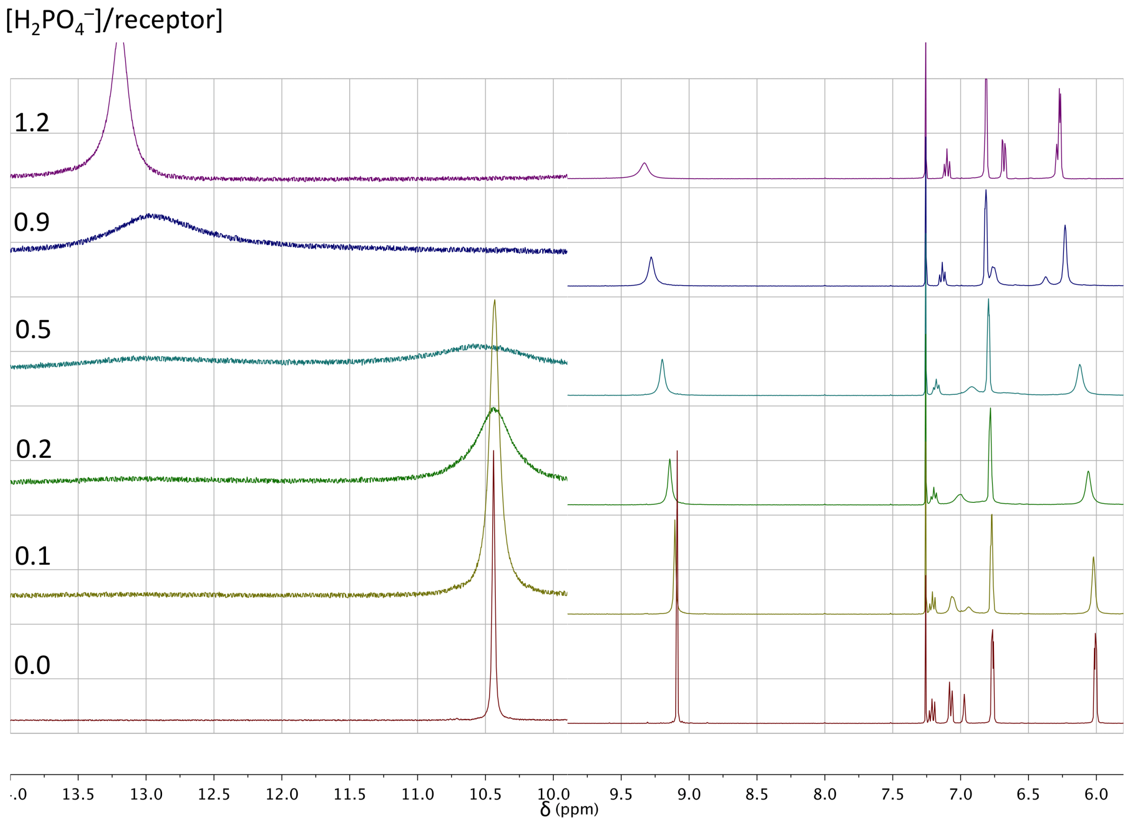


**Fig. SI-9** Stacked $^{1}$H NMR spectra corresponding to the titration of **4b** with $TBH_2PO_4$ in $CDCl_3$.

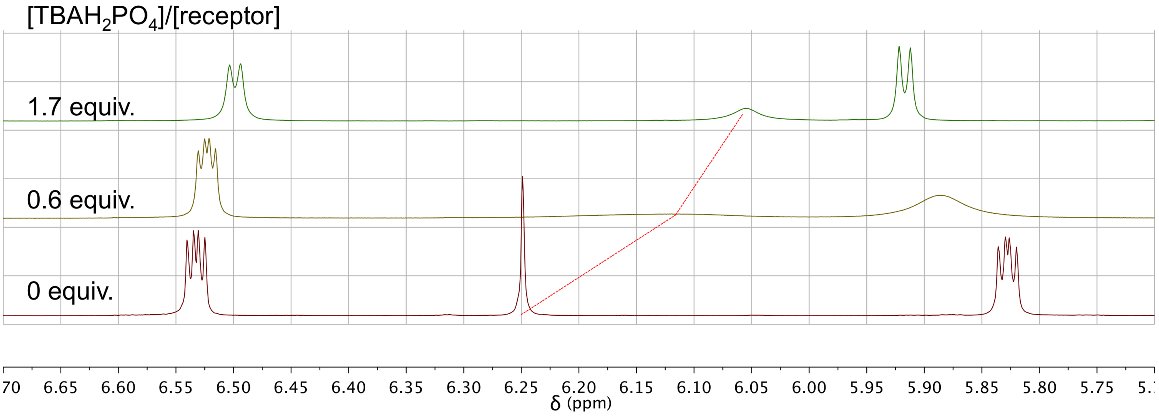


**Fig. SI-10** Stacked $^{1}$H NMR spectra of receptor **7b** (15.6 mM) with varying quantities of $H_2PO_4^-$ recorded in $CDCl_3$:DMSO-$d_6$ (8:1). Dashed lines show changes in the chemical shifts for the aromatic hydrogen atoms of the benzene ring of receptor **7b** ($\Delta\delta$ = -0.19 ppm).

**UV-Vis Spectroscopy**

All binding studies reported in Table 2 were carried out using commercially available Fisher Scientific $CHCl_3$ containing approximately 0.75% ethanol as a preservative and commercially available Fisher Scientific $CH_3OH$. All anions were used in the form of their commercially available tetra-*n*-butylammonium ($TBA^+$) salts.

In the titrations carried out with intention of determining binding affinities, the concentration of the receptor in question was kept constant. This was achieved by adjusting the guest (anion) concentration within stock host (receptor) solutions. This allowed the binding studies to be carried out without having to make mathematical corrections due to change in total receptor concentration.

$$\text{receptor} \quad + \quad \text{anion} \quad \rightarrow \quad \text{receptor}\cdot\text{anion}$$

$$K_a = \frac{[\text{receptor} \cdot \text{anion}]}{[\text{receptor}] * [\text{anion}]}$$

In the wavelength range of 250-400 nm:

$\varepsilon_{\lambda,\text{receptor}} \neq \varepsilon_{\lambda,\text{receptor}\cdot\text{anion}}$

$\varepsilon_{\lambda,\text{anion}} = 0$

Throughout the titration

$[\text{receptor}]_o = [\text{receptor}] + [\text{receptor}\cdot\text{anion}]$

$[\text{anion}]_o = [\text{anion}] + [\text{receptor}\cdot\text{anion}]$

The following equation was used to determine the binding affinities:

$$\Delta A = \frac{1 + aK + xK - \sqrt{-4axK^2 + (-1 - aK - xK)^2}}{2K} \Delta\varepsilon$$

where,

$\Delta$A (measured change in absorbance relative to the initial solution) is the y variable;

x is concentration of guest (in the form of its $TBA^+$ salt) added;

$a$ is the initial concentration of receptor (remains constant over the course of titration);

$\Delta\varepsilon$ is the extinction coefficient difference between the free receptor and the receptor-guest complex;

*K* ($K_a$) is the binding affinity of the receptor for the guest in question.

OriginPro 9.0 was used to fit the data in a non-linear fashion in accord with the above equation. Iterations (at a fixed $a$ value, by changing $\Delta\varepsilon$ and *K*) were performed until an acceptable level of convergence was reached (chi-square tolerance value, $1 \times 10^{-9}$). Fig. SI-11 through Fig. SI-19 show the UV-Vis spectra and the binding curve fittings for receptors **3b** and **7b**.

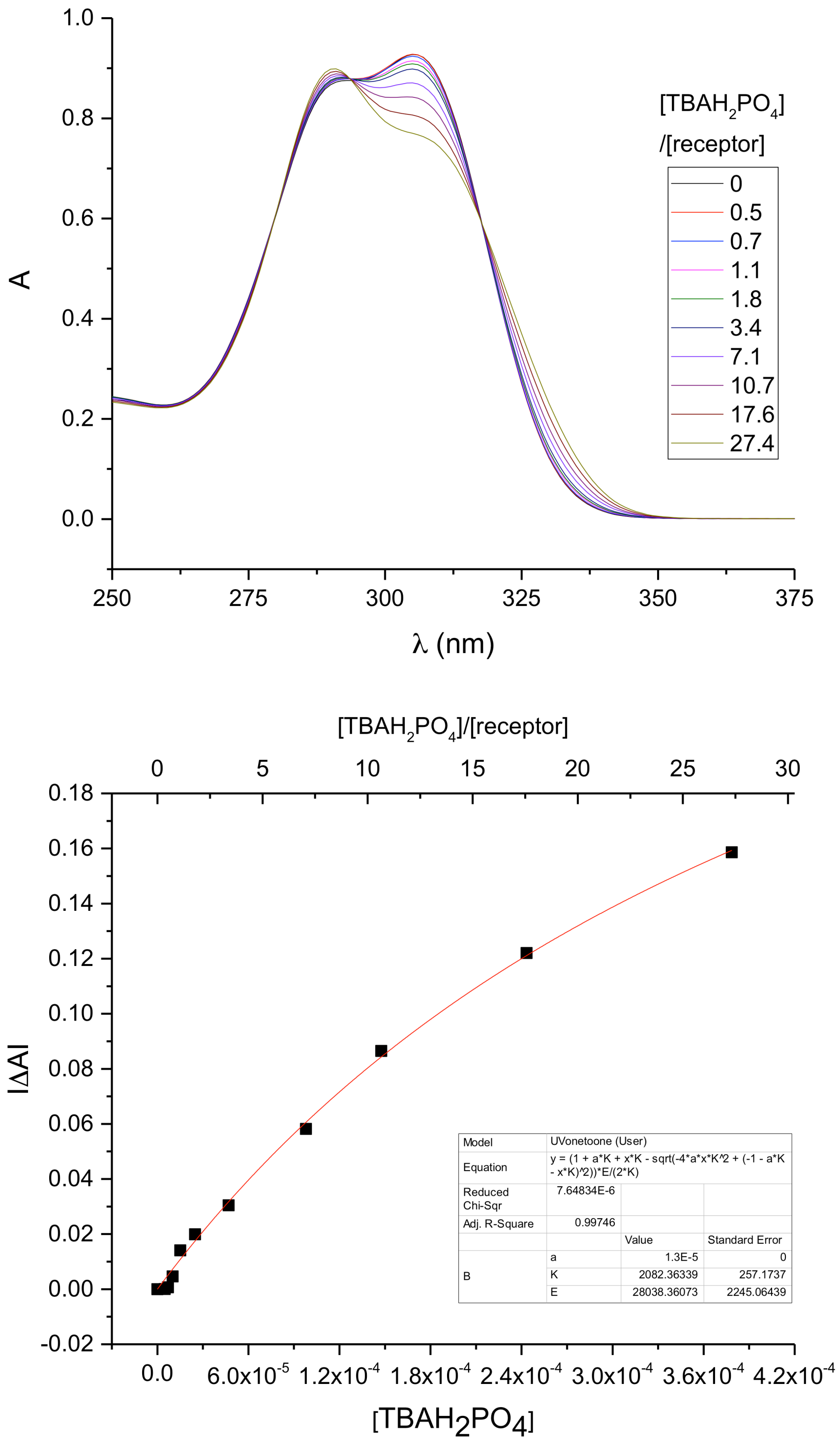


**Fig. SI-11** Top: Stacked UV-Vis spectra for titration of **3b** ($1.3 \times 10^{-5}$ M) with $TBAH_2PO_4$ ($4.1 \times 10^{-3}$ M) in $CHCl_3$ containing 3% (v/v) $CH_3OH$. Bottom: Binding curve and fit generated from the titration data at 305 nm. $K_a = (2.1 \pm 0.3) \times 10^3$ $M^{-1}$.

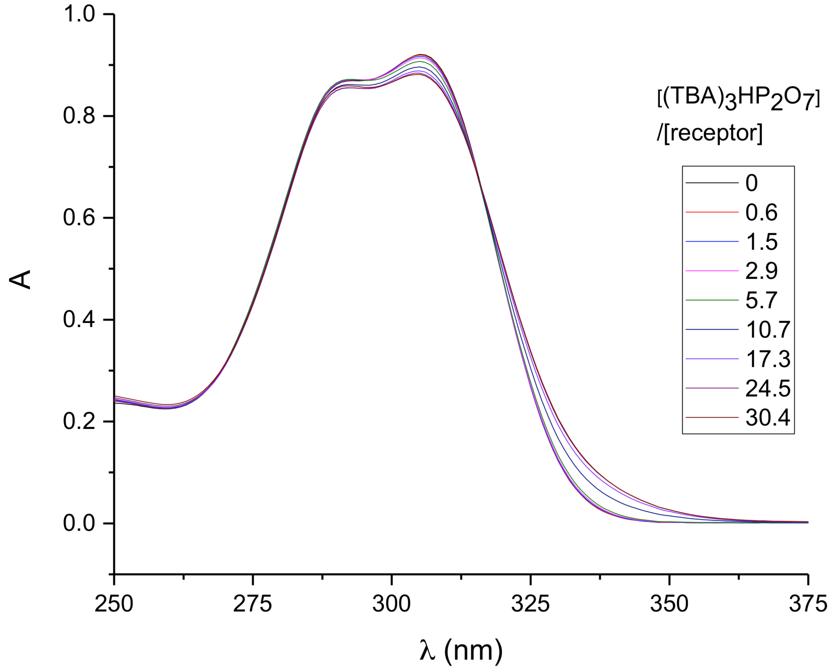


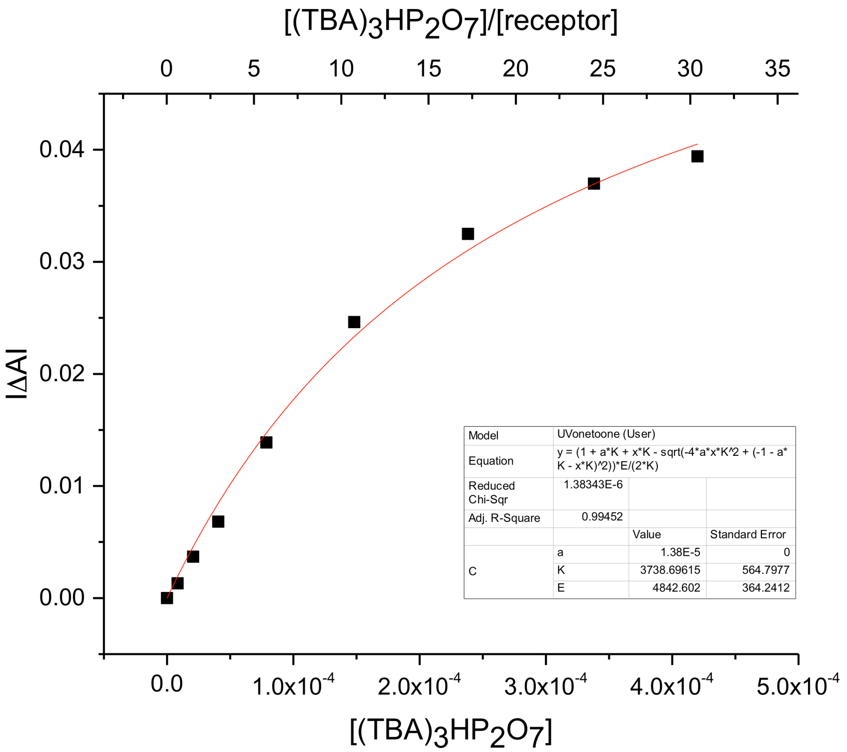


**Fig. SI-12** Top: Stacked UV-Vis spectra for titration of **3b** ($1.3 \times 10^{-5}$ M) with $(TBA)_3HP_2O_7$ ($1.3 \times 10^{-3}$ M) in $CHCl_3$ containing 3% (v/v) $CH_3OH$. Bottom: Binding curve and fit generated from the titration data at 305 nm. $K_a = (3.8 \pm 0.6) \times 10^3$ $M^{-1}$.

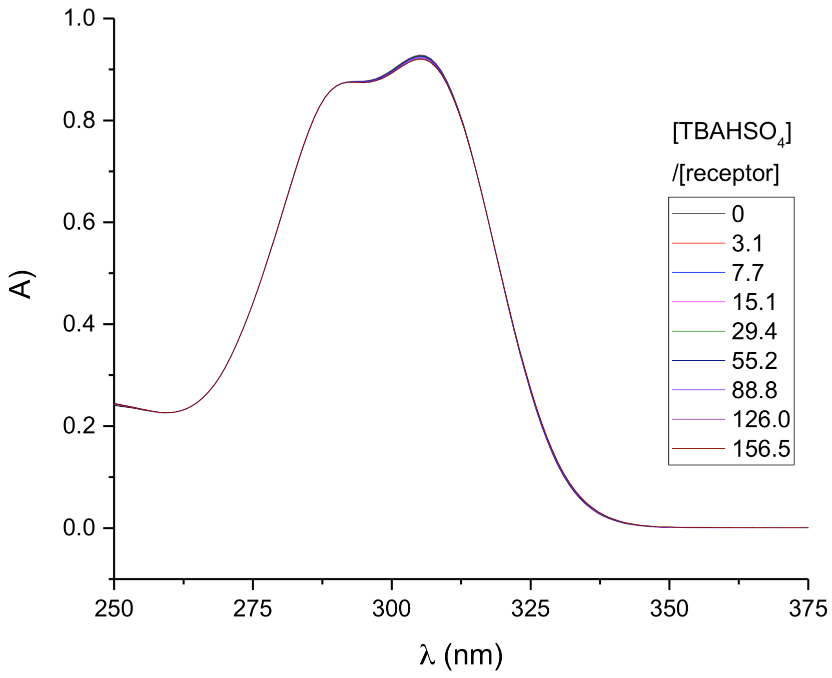


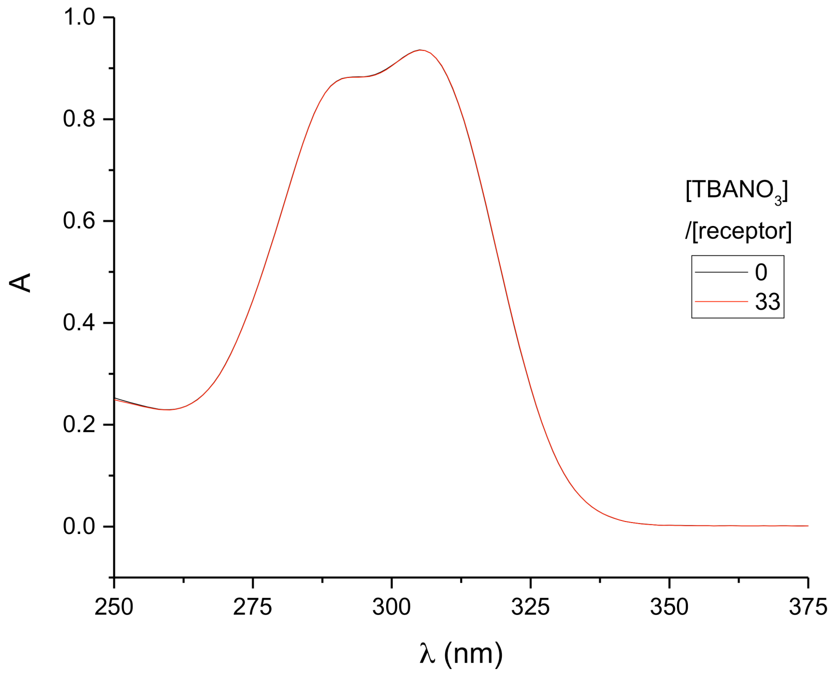


**Fig. SI-13** Top: Stacked UV-Vis spectra for titration of **3b** ($1.3 \times 10^{-5}$ M) with $TBAHSO_4$ ($6.5 \times 10^{-3}$ M) in $CHCl_3$ containing 3% (v/v) $CH_3OH$. Bottom: Stacked UV-Vis spectra of **3b** ($1.3 \times 10^{-5}$ M) with and without $TBANO_3$ ($4.6 \times 10^{-4}$ M) in $CHCl_3$ containing 3% (v/v) $CH_3OH$.

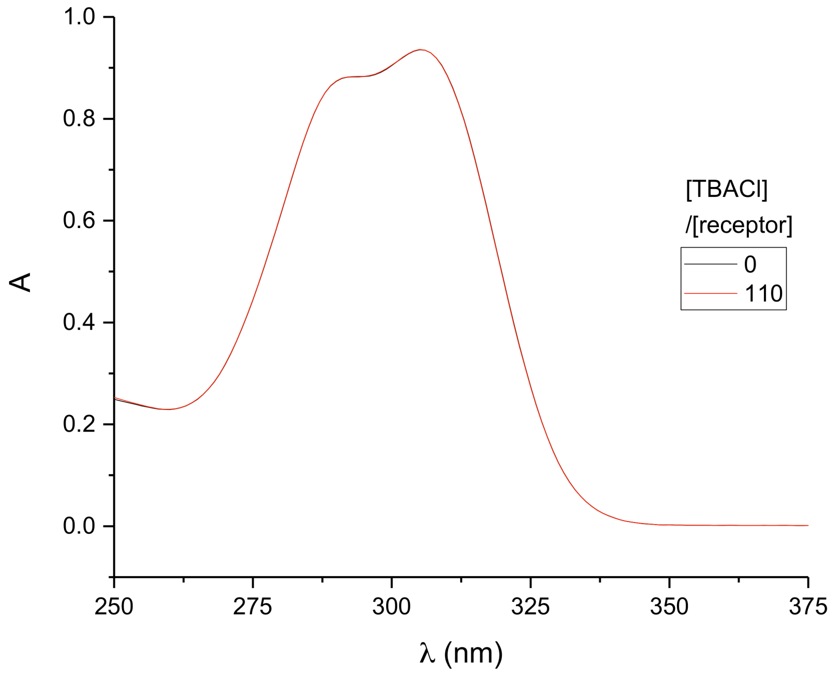


**Fig. SI-14** Stacked UV-Vis spectra of **3b** ($1.3 \times 10^{-5}$ M) with and without TBACl ($1.5 \times 10^{-3}$ M) in $CHCl_3$ containing 3% (v/v) $CH_3OH$.

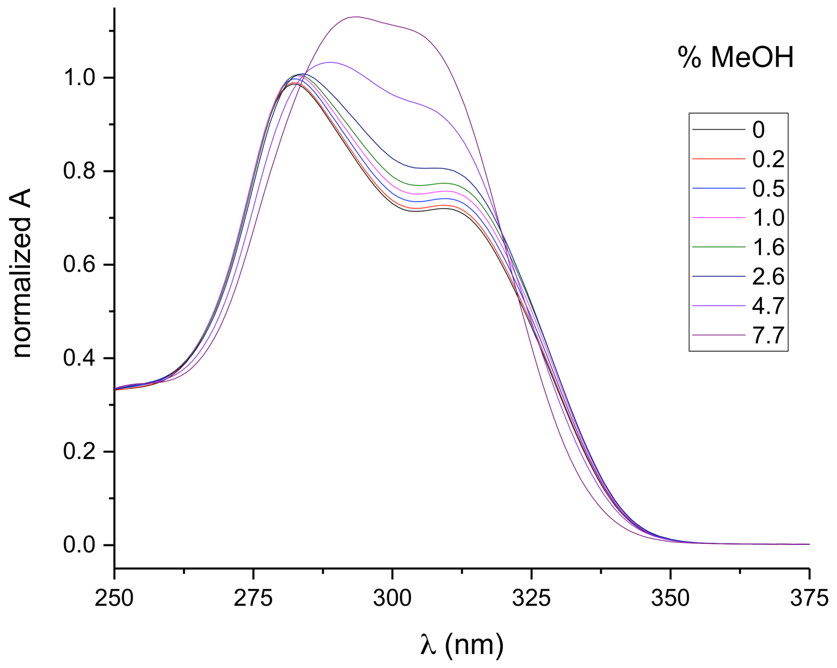


**Fig. SI-15** Stacked UV-Vis spectra of **7b** solubilized by 2.75 equivalent $TBAH_2PO_4$ salt in neat $CHCl_3$ (0% added $CH_3OH$) and increasing percentages of added $CH_3OH$. Spectra are normalized to eliminate the effect of dilution.

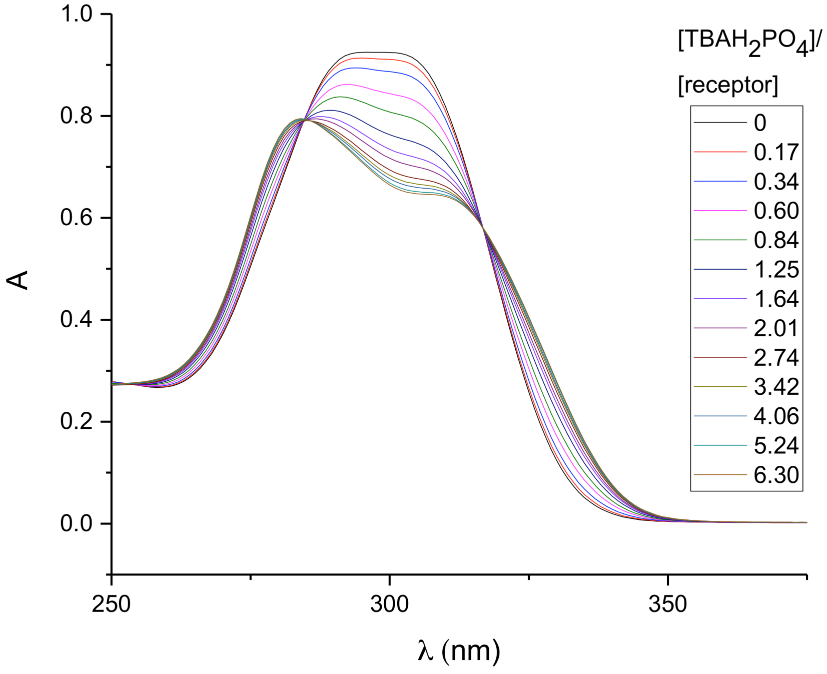


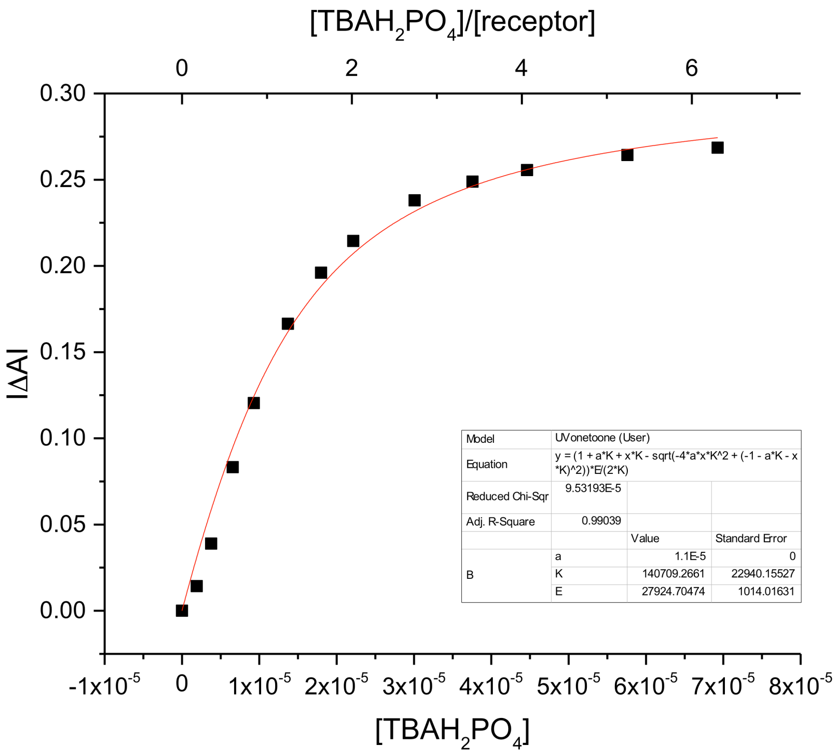


**Fig. SI-16** Top: Stacked UV-Vis spectra for titration of **7b** ($1.1 \times 10^{-5}$ M) with $TBAH_2PO_4$ ($2.9 \times 10^{-4}$ M) in $CHCl_3$ containing 3% (v/v) $CH_3OH$. Bottom: Binding curve and fit generated from the titration data at 302 nm. $K_a = (1.4 \pm 0.2) \times 10^5\ M^{-1}$.

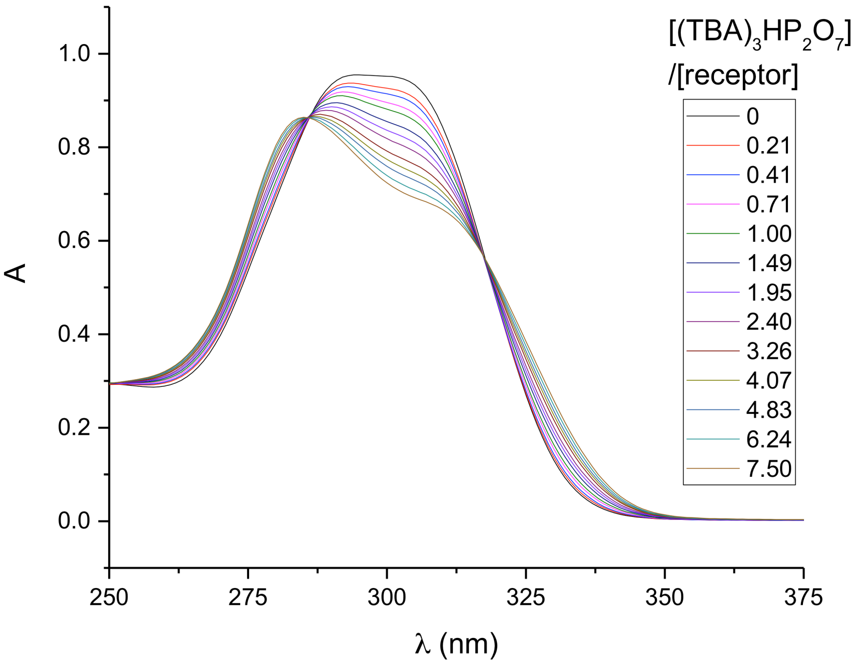


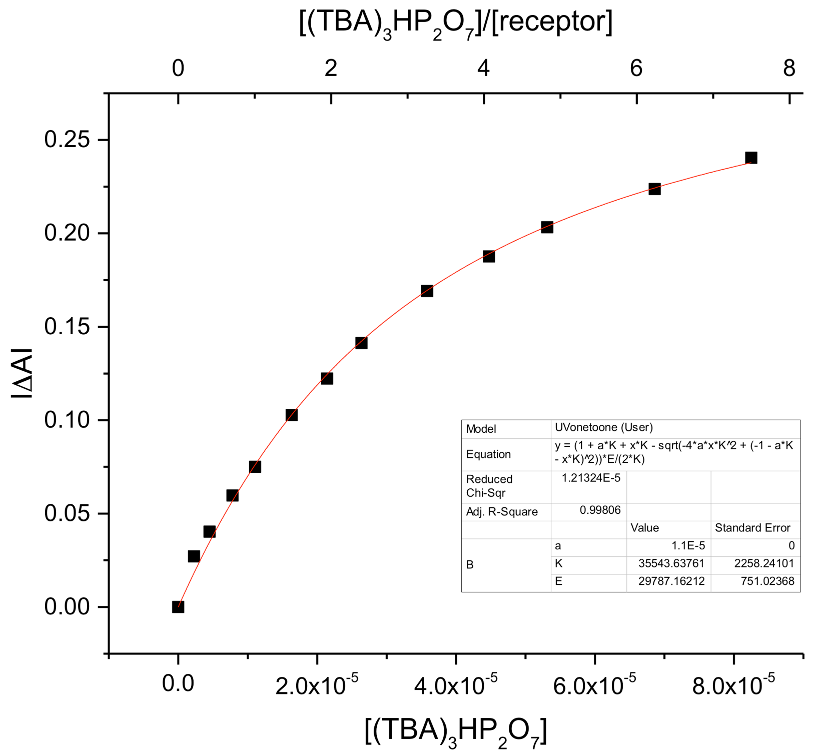


**Fig. SI-17** Top: Stacked UV-Vis spectra for titration of **7b** ($1.1 \times 10^{-5}$ M) with $(TBA)_3HP_2O_7$ ($2.9 \times 10^{-4}$ M) in $CHCl_3$ containing 3% (v/v) $CH_3OH$. Bottom: Binding curve and fit generated from the titration data at 302 nm. $K_a = (3.6 \pm 0.2) \times 10^4\ M^{-1}$.

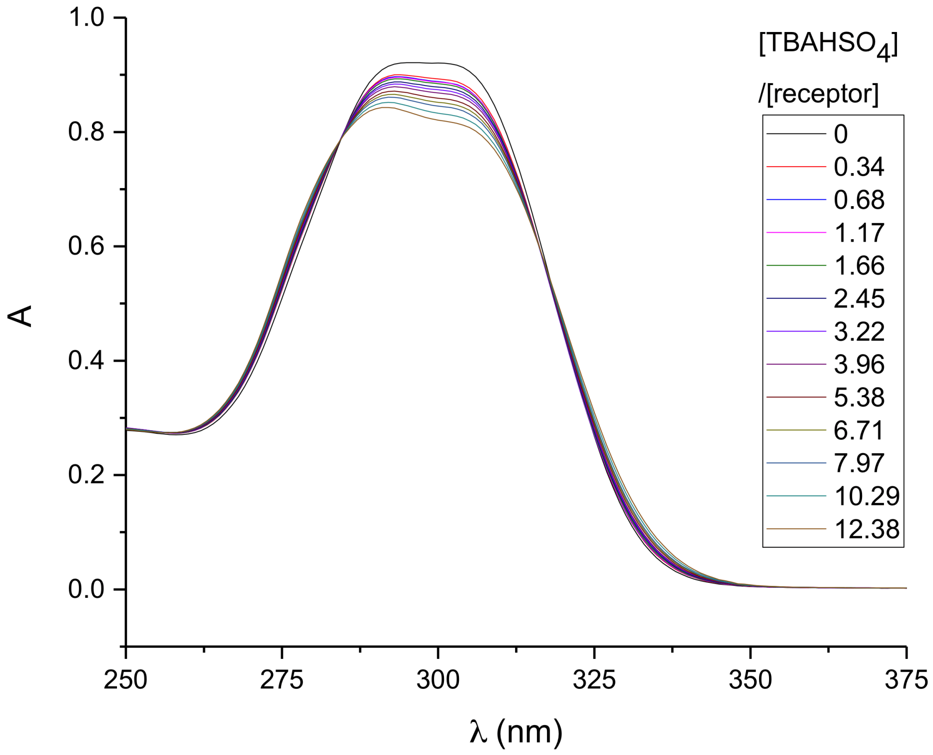


**Fig. SI-18** Stacked UV-Vis spectra for titration of **7b** ($1.1 \times 10^{-5}$ M) with $TBAHSO_4$ ($5.7 \times 10^{-4}$ M) in $CHCl_3$ containing 3% (v/v) $CH_3OH$. No saturation behaviour is observed.

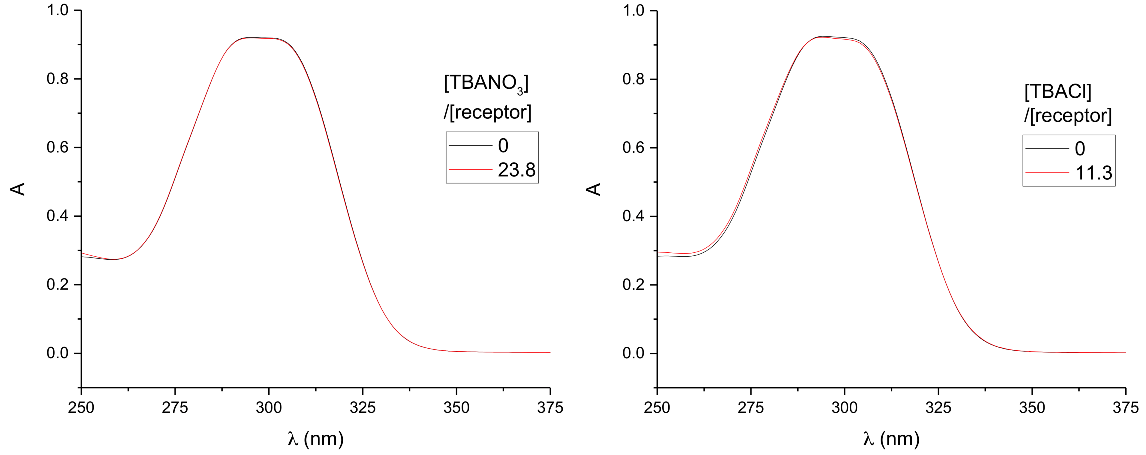


**Fig. SI-19** Left: Stacked UV-Vis spectra for titration of **7b** ($1.1 \times 10^{-5}$ M) with $TBANO_3$ ($2.4 \times 10^{-4}$ M) in $CHCl_3$ containing 3% (v/v) $CH_3OH$. Right: Stacked UV-Vis spectra for titration of **7b** ($1.1 \times 10^{-5}$ M) with TBACl ($1.2 \times 10^{-4}$ M) in $CHCl_3$ containing 3% (v/v) $CH_3OH$.

**Cyclic Voltammetry**

For the titration of **6b** with $TBAH_2PO_4$ in $CH_3CN$ containing 10% (v/v) DMF, two chemical equilibria can be written as follows.

$$\mathbf{6b}\ (s) \rightleftharpoons \mathbf{6b}\ (org) \qquad K_{sp}$$

$$\mathbf{6b}\ (org) + H_2PO_4^-(org) \rightleftharpoons \mathbf{6b}\cdot H_2PO_4^-\ (org) \qquad K_a$$

The concentration of **6b** remains constant during the titration when there is still insoluble **6b**. This approximation is especially valid for the earlier data points.

$$[\mathbf{6b}] = K_{sp}$$

The peak current ($i_p$) is a function of concentration as defined by the following equation:

$$i_p = 2.69 \times 10^5\, n^{3/2} v^{1/2} A D C$$
$$i_p = \kappa\, C$$

Where n is the number of electrons transferred, v is the scan rate, A is the electrode surface area, C is the concentration and D is the diffusion coefficient. In the simplified equation, $\kappa$ is a derived parameter that is held constant.

$$i_{p,o} = \kappa_{\mathbf{6b}}.[\mathbf{6b}]$$
$$i_p = \kappa_{\mathbf{6b}}.[\mathbf{6b}] + \kappa_{complex}.[\mathbf{6b}\cdot H_2PO_4^-]$$
$$\Delta i_p = \kappa_{complex}.[\mathbf{6b}\cdot TBAH_2PO_4^-]$$
$$K_a = \frac{[\mathbf{6b}\cdot H_2PO_4^-]}{[\mathbf{6b}].[H_2PO_4^-]}$$

$$K_a = \frac{\Delta i_p/\kappa_{complex}}{\left(\frac{i_{p,o}}{\kappa_{6b}}\right).[\mathrm{H_2PO_4}^-]}$$

$[\mathbf{6b}\cdot H_2PO_4^-]_{max}$ = 1 mM

$i_{p,max} = \kappa_{complex}$ $[\mathbf{6b}\cdot H_2PO_4^-]_{max}$

it is assumed that all of the initial sample of **6b** is solubilized upon formation of complex ($\mathbf{6b}\cdot H_2PO_4^-$) and $\kappa_{complex} = \kappa_{\mathbf{6b}}$

For the calculations,

$[\mathbf{6b}]_{sat} = i_{p,max}/[\mathbf{6b}]_{max}$

$[\mathbf{6b}\cdot H_2PO_4^-] = \Delta i_p\ ([\mathbf{6b}\cdot H_2PO_4^-]/\ i_{p,max})$

$[H_2PO_4^-] = [H_2PO_4^-]_o - [\mathbf{6b}\cdot H_2PO_4^-]$

A plot of $\Delta i_p$ against added $H_2PO_4^-$ ($[H_2PO_4^-]_o$) is shown in Fig. SI-20 and the calculated equilibrium composition is presented in Table SI-1.

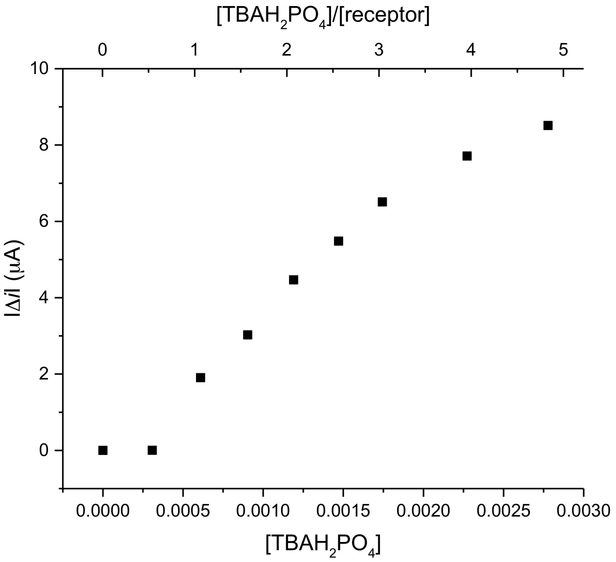


**Fig. SI-20** The change in anodic peak current at around 600 mV is plotted against concentration of $TBAH_2PO_4$ added. Titration voltammograms are presented in Fig. 8 of the main text

**Table SI-1** Excel spreadsheet used to calculate the *K* value corresponding to the formation of **6b**·$H_2PO_4^-$.

| Experimental conditions | | Unit | | Calculated | | Unit | |
|---|---|---|---|---|---|---|---|
| [**6b**]$_{max}$ | 1.0E-03 | M | | [**6b**]$_{sat}$ | 3.50E-04 | M | |
| $i_{p,max}$ | 13.1 | uA | | | | | |
| | | | | | | | |
| Experimental | Calculated | | | | | | |
| $[H_2PO_4^-]_o$ | I*i*I (μA) | Δ*i* | [**6b**·$H_2PO_4^-$] | [**6b**] | [**6b**]$_t$ | $[H_2PO_4^-]$ | $K_a$ |
| 0.00E+00 | 4.589 | 0.00E+00 | 0.00E+00 | 3.50E-04 | 3.50E-04 | 0.00E+00 | n/a |
| 3.09E-04 | 4.592 | 3.00E-03 | 2.29E-07 | 3.50E-04 | 3.51E-04 | 3.08E-04 | outlier |
| 6.10E-04 | 6.495 | 1.91E+00 | 1.45E-04 | 3.50E-04 | 4.96E-04 | 4.64E-04 | 8.95E+02 |
| 9.04E-04 | 7.615 | 3.03E+00 | 2.31E-04 | 3.50E-04 | 5.81E-04 | 6.73E-04 | 9.80E+02 |
| 1.19E-03 | 9.057 | 4.47E+00 | 3.41E-04 | 3.50E-04 | 6.91E-04 | 8.49E-04 | 1.15E+03 |
| 1.47E-03 | 10.07 | 5.48E+00 | 4.18E-04 | 3.50E-04 | 7.69E-04 | 1.05E-03 | 1.14E+03 |
| 1.74E-03 | 11.1 | 6.51E+00 | 4.97E-04 | 3.50E-04 | 8.47E-04 | 1.25E-03 | 1.14E+03 |
| 2.27E-03 | 12.3 | 7.71E+00 | 5.89E-04 | 3.50E-04 | 1.00E-03 | 1.68E-03 | 9.98E+02 |
| 2.78E-03 | 13.1 | 8.51E+00 | 6.50E-04 | 3.50E-04 | 1.00E-03 | 2.13E-03 | 8.72E+02 |
| | | | | | | $K_{a,av}$ | 1.02E+03 |
| | | | | | | STD | 1.17E+02 |
| | | | | | | %std | 1.15E-01 |
| Note: $i_{p,max}$ input in the Excel sheet has an influence on calculated $K_a$ value for **6b**·$H_2PO_4^-$ complex formation. | | | | | | | |

A control experiment was performed using ferrocene under the same experimental conditions as used for the study **6b**. It is important to note that there is no anodic peak current increase observed in the experiment carried with ferrocene. However, upon inspection of cyclic voltammograms corresponding to this titration, a new reduction signal at 280 mV is seen (*cf*. Fig. SI-21). This new reduction signal is ascribed to the reduction of $H^+$ that originates from the $H_2PO_4^-$ anion. Consistent with this conclusion is the reduction in the intensity of the signal observed when pyridine is added to the medium (*cf*. Fig. SI-22).

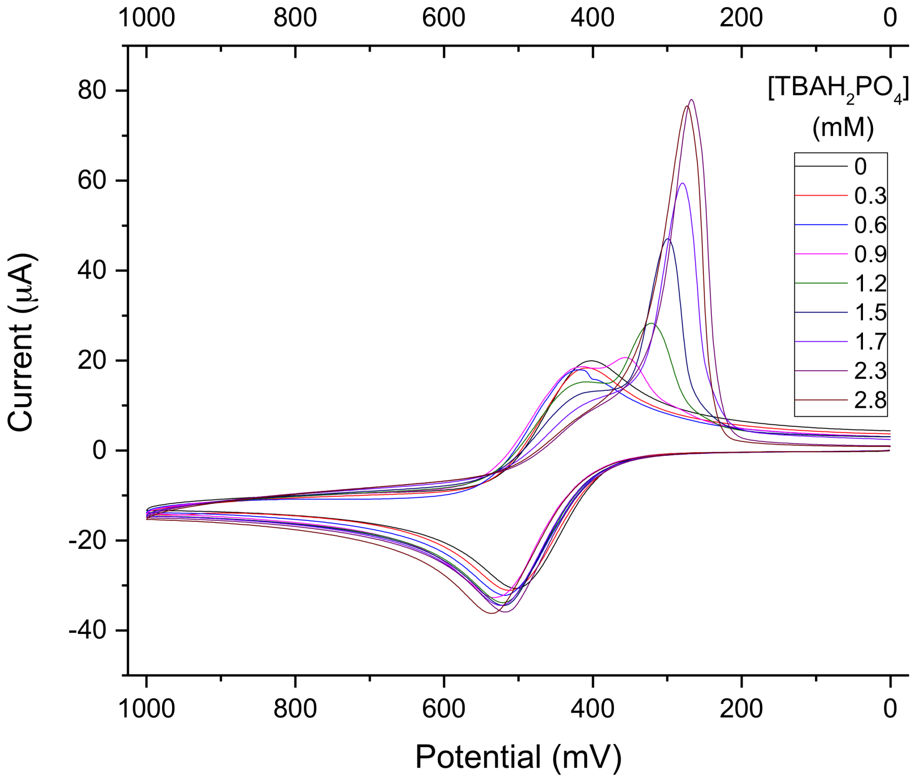


**Fig. SI-21** Stacked cyclic voltammogram of 1.0 mM ferrocene in $CH_3CN$ containing 10% (v/v) DMF (black line). This solution was titrated with $TBAH_2PO_4$ until the $TBAH_2PO_4$ concentration reached 2.8 mM. $TBAPF_6$ (0.1 M) was used as the supporting electrolyte. Glassy carbon was used as the working electrode, a Pt wire as the counter electrode, and a Ag/AgCl couple as the reference electrode.

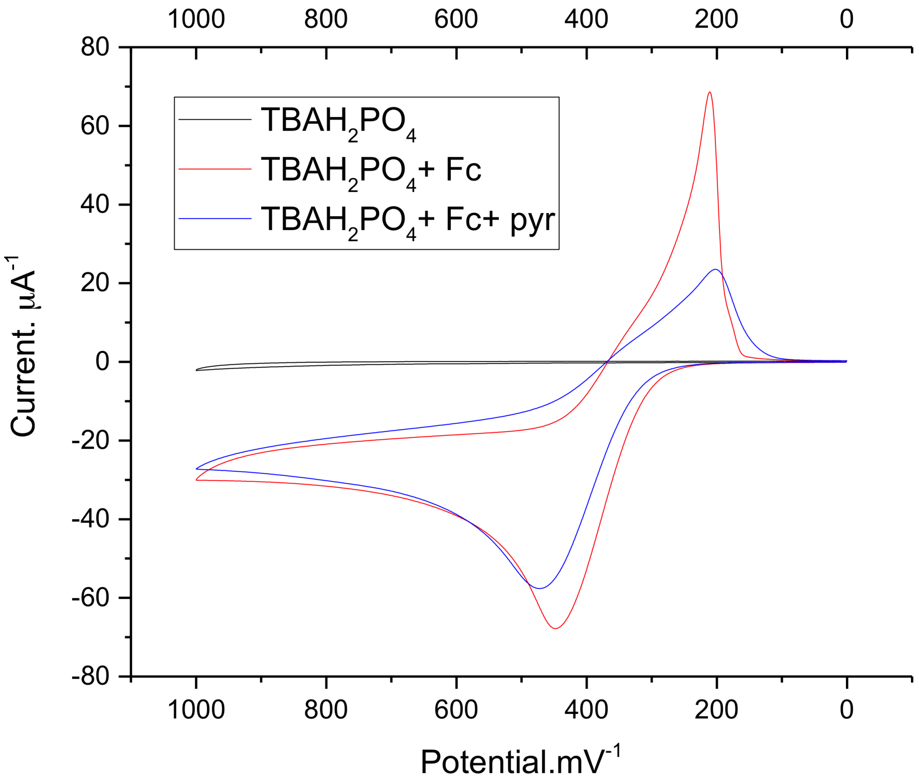


**Fig. SI-22** Stacked cyclic voltammogram of $TBAH_2PO_4$ (15 mM) in $CH_3CN$ containing 10% (v/v) DMF (black line). Solid ferrocene was added to achieve a 2.0 mM concentration (red line). To this solution, 0.2 mL pyridine was added (blue line). $TBAPF_6$ (0.1 M) was used as the supporting electrolyte. Glassy carbon was used as the working electrode, a Pt wire as the counter electrode, and a Ag/AgCl couple as the reference electrode.

### X-ray Diffraction Analyses

The X-ray diffraction data collection and refinements for the crystal structures of **6b**, **7b**, [**3b**·$(TBA)_3H_2P_2O_7]_2$, **4b**·$TBAH_2PO_4$, and [**7b**·$TBAH_2PO_4]_2$ may be obtained from the Cambridge Crystallographic Data Centre (CCDC) by reference to CCDC numbers 1444557-1444561. Some experimental details and relevant data tables for the refinements in question are given below.

### Experimental Details for X-ray Diffraction Analysis

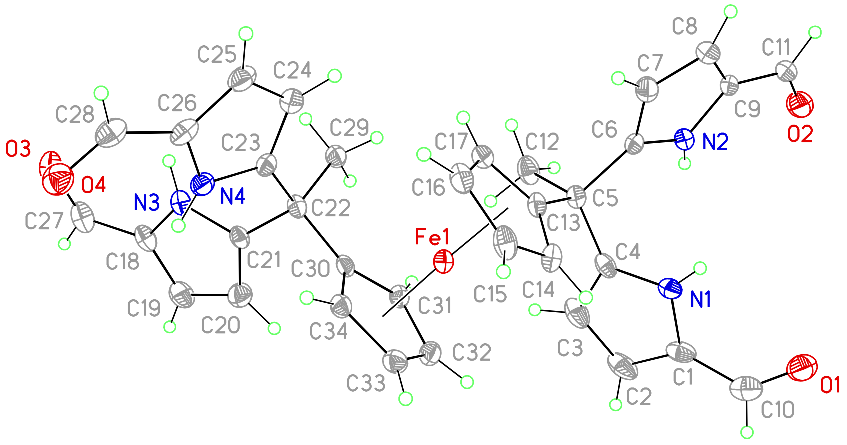


X-ray Experimental for **6b** $(C_{17}H_{15}N_2O)_2Fe$ – 2 $C_3H_7NO$: Crystals grew as clear, yellow prisms by slow evaporation from dimethylformamide with a small amount of dichloromethane. The data crystal had approximate dimensions; 0.27 x 0.20 x 0.09 mm. The data were collected at -140 °C on a Nonius Kappa CCD diffractometer using a Bruker AXS Apex II detector and a graphite monochromator with MoKα radiation (λ = 0.71073 Å). Reduced temperatures were maintained by use of an Oxford Cryosystems 600 low-temperature device. A total of 1110 frames of data were collected using ω and ϕ-scans with a scan range of 1.1° and a counting time of 43 seconds per frame. Details of crystal data, data collection and structure refinement are listed in Table ESI-2. Data reduction was performed using SAINT V8.27B.[2] The structure was solved by direct methods using SUPERFLIP[3] and refined by full-matrix least-squares on $F^2$

with anisotropic displacement parameters for the non-H atoms using SHELXL-2013.[4] Structure analysis was aided by use of the programs PLATON98[5] and WinGX.[6] The hydrogen atoms bound to carbon atoms were calculated in idealized positions. The hydrogen atoms on the pyrrole nitrogen atoms were observed in a ΔF map and refined with isotropic displacement parameters.

The function, $\Sigma w(|F_o|^2 - |F_c|^2)^2$, was minimized, where $w = 1/[(\sigma(F_o))^2 + (0.0331*P)^2 + (2.7996*P)]$ and $P = (|F_o|^2 + 2|F_c|^2)/3$. $R_w(F^2)$ refined to 0.111, with R(F) equal to 0.0471 and a goodness of fit, S, = 1.01. Definitions used for calculating R(F),$R_w(F^2)$ and the goodness of fit, S, are given below.[7] The data were checked for secondary extinction but no correction was necessary. Neutral atom scattering factors and values used to calculate the linear absorption coefficient are from the International Tables for X-ray Crystallography (1992).[8] All figures were generated using SHELXTL/PC.[9] Tables of positional and thermal parameters, bond lengths and angles, torsion angles and figures may be obtained from the CCDC by referencing CCDC number 1444557.

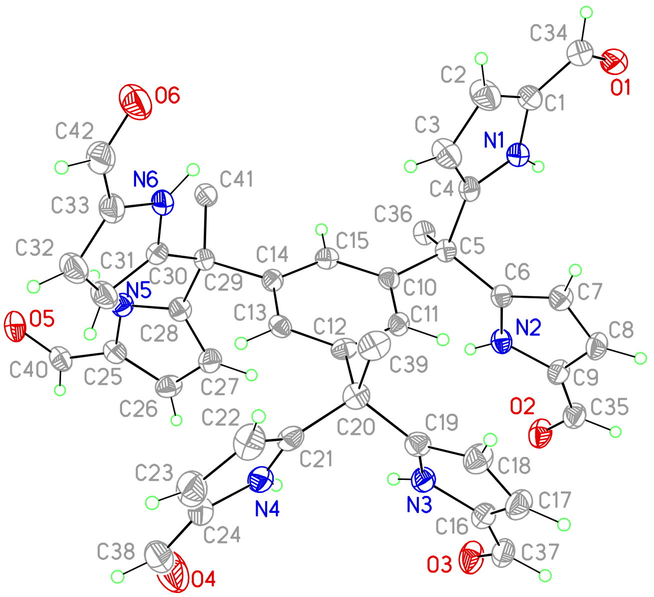


X-ray Experimental for **7b** $C_{42}H_{36}N_6O_6$ - 2 $CHCl_3$: Crystals grew as clusters of yellow prisms by slow evaporation from chloroform, methanol and dimethylformamide. The data crystal was cut from a larger crystal and had approximate dimensions; 0.30 x 0.23 x 0.15 mm. The data were collected on a

Rigaku SCX-Mini diffractometer with a Mercury 2+ CCD using a graphite monochromator with MoK$\alpha$ radiation ($\lambda$ = 0.71073 Å). A total of 1080 frames of data were collected using $\omega$-scans with a scan range of 0.5° and a counting time of 25 seconds per frame. The data were collected at 100 K using a Rigaku XStream low temperature device. Details of crystal data, data collection and structure refinement are listed in Table ESI-3. Data reduction was performed using the Rigaku Americas Corporation's Crystal Clear version 1.40.[10] The structure was solved by direct methods using SIR2004[11] and refined by full-matrix least-squares on $F^2$ with anisotropic displacement parameters for the non-H atoms using SHELXL-97.[4] Structure analysis was aided by use of the programs PLATON98[5] and WinGX.[6] The hydrogen atoms were calculated in ideal positions with isotropic displacement parameters set to 1.2xUeq of the attached atom (1.5xUeq for methyl hydrogen atoms).

A molecule of chloroform was disordered. The disorder resulted in two principal conformations. The disorder was modelled by assigning the variable x to the site occupancy for one component and (1-x) to the site occupancy of the alternate component. The variable x was refined while restraining the geometry of the two components to be equivalent. A common isotropic displacement parameter was refined while refining x. In this way, the major component of the disordered solvent molecule consisting of atoms, Cl4, Cl5, Cl6 and C1b refined to a site occupancy of 53(1)%. The non-H atoms of both components were ultimately refined anisotropically with their displacement parameters restrained to be approximately isotropic.

The function, $\Sigma w(|F_o|^2 - |F_c|^2)^2$, was minimized, where $w = 1/[(\sigma(F_o))^2 + (0.086^*P)^2 + (3.5129^*P)]$ and $P = (|F_o|^2 + 2|F_c|^2)/3$. $R_w(F^2)$ refined to 0.2593, with R(F) equal to 0.0937 and a goodness of fit, S, = 1.16. Definitions used for calculating R(F), $R_w(F^2)$ and the goodness of fit, S, are given below.[7] The data were checked for secondary extinction effects but no correction was necessary. Neutral atom scattering factors and values used to calculate the linear absorption coefficient are from the International Tables for X-ray Crystallography (1992).[8] All figures were generated using SHELXTL/PC.[9] Tables of positional and thermal

parameters, bond lengths and angles, torsion angles and figures may be obtained from the CCDC by referencing CCDC number 1444558.

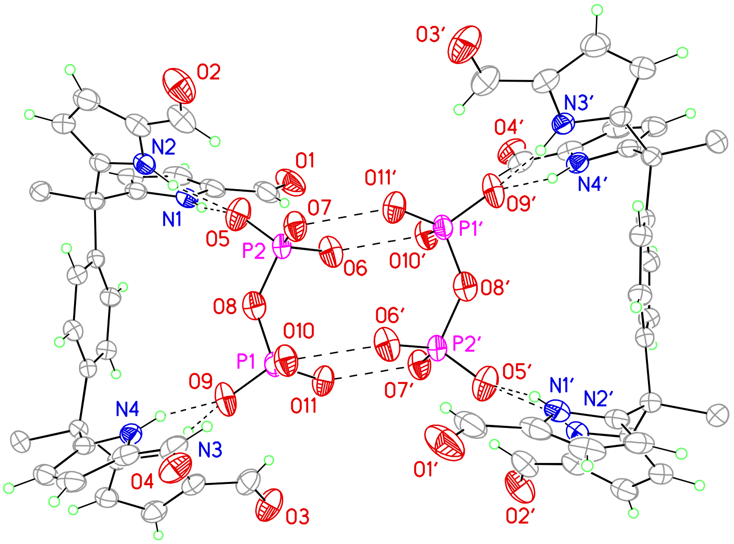


X-ray Experimental for **3b·**(TBA)$_2$H$_2$P$_2$O$_7$ $C_{30}H_{26}N_4O_4$ - 2 $C_{16}H_{36}N^{1+}$ $H_2P_2O_7^{2-}$ ½ $CH_2Cl_2$: Crystals grew as thin, colourless prisms by layering a solution of **3b**·(TBA)$_3$HP$_2$O$_7$ in $CH_2Cl_2$ with *n*-pentane. The data crystal had approximate dimensions; 0.16 x 0.11 x 0.06 mm. The data were collected on an Agilent Technologies SuperNova Dual Source diffractometer using a μ-focus Cu Kα radiation source (λ = 1.5418Å) with collimating mirror monochromators. A total of 850 frames of data were collected using ω-scans with a scan range of 0.5° and a counting time of 7 seconds per frame using a detector offset of +/- 41.1° and a counting time of 25 seconds per frame using a detector offset of +/- 108.3°. The data were collected at 100 K using an Oxford Cryostream low temperature device. Details of crystal data, data collection and structure refinement are listed in Table ESI-4. Data collection, unit cell refinement and data reduction were performed using Agilent Technologies CrysAlisPro V 1.171.37.31.[12] The structure was solved by direct methods using SIR97[13] and refined by full-matrix least-squares on $F^2$ with anisotropic displacement parameters for the non-H atoms using SHELXL-2013.[4] Structure analysis was aided by use of the programs PLATON98[5] and WinGX.[6] The hydrogen atoms

were calculated in ideal positions with isotropic displacement parameters set to 1.2xUeq of the attached atom (1.5xUeq for methyl hydrogen atoms.

Compound **3b** and the pyrophosphate form H-bound dimers across a crystallographic inversion centre located at ½, ½, ½. The pyrophosphate dianion was H-bound to itself across this centre. Reliable coordinates for the hydrogen atoms bound to the pyrophosphate could not be located in a difference electron density map. These hydrogen atoms were not included in the final refinement model.

A molecule of dichloromethane was found to be badly disordered around a crystallographic inversion centre at 0, ½, ½. Attempts to model the disorder were unsatisfactory. The contributions to the scattering factors due to this solvent molecule were removed by use of the utility SQUEEZE[14] in PLATON98. PLATON98 was used as incorporated in WinGX.

The function, $\Sigma w(|F_o|^2 - |F_c|^2)^2$, was minimized, where $w = 1/[(\sigma(F_o))^2 + (0.0827*P)^2 + (5.3705*P)]$ and $P = (|F_o|^2 + 2|F_c|^2)/3$. $R_w(F^2)$ refined to 0.171, with R(F) equal to 0.0603 and a goodness of fit, S, = 1.03. Definitions used for calculating R(F), $R_w(F^2)$ and the goodness of fit, S, are given below.[7] The data were checked for secondary extinction effects but no correction was necessary. Neutral atom scattering factors and values used to calculate the linear absorption coefficient are from the International Tables for X-ray Crystallography (1992).[8] All figures were generated using SHELXTL/PC.[9] Tables of positional and thermal parameters, bond lengths and angles, torsion angles and figures may be obtained from the CCDC by referencing CCDC number 1444559.

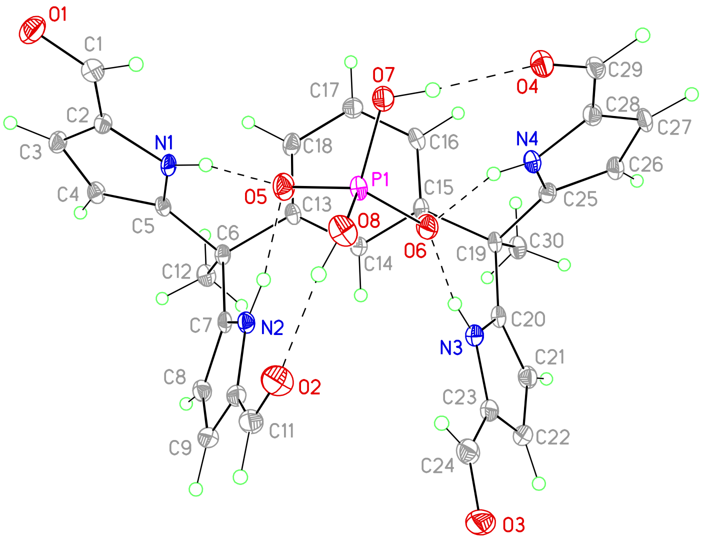


X-ray Experimental for complex **4b**·TBA$H_2PO_4$ ($C_{30}H_{26}N_4O_4$)($C_{16}H_{36}N$)$^{1+}$ $O_2P(OH)_2^{1-}$ -1/2 $CHCl_3$: Crystals grew as yellow plates by layering a solution of **4b**·TBA$H_2PO_4$ in $CHCl_3$ with *n*-pentane.The data crystal had approximate dimensions; 0.18 x 0.07 x 0.05 mm. The data were collected on an Agilent Technologies SuperNova Dual Source diffractometer using a μ-focus Cu Kα radiation source (λ = 1.5418Å) with collimating mirror monochromators. A total of 1659 frames of data were collected using ω-scans with a scan range of 1° and a counting time of 9 seconds per frame with a detector offset of +/- 40.8° and 20 seconds per frame with a detector offset of +/- 108.3°. The data were collected at 100 K using an Oxford Cryostream low temperature device. Details of crystal data, data collection and structure refinement are listed in Table ESI-5. Data collection, unit cell refinement and data reduction were performed using Agilent Technologies CrysAlisPro V 1.171.37.31.[12] The structure was solved by direct methods using SuperFlip[15] and refined by full-matrix least-squares on $F^2$ with anisotropic displacement parameters for the non-H atoms using SHELXL-2013.[4] Structure analysis was aided by use of the programs PLATON98[5] and WinGX.[6] The hydrogen atoms bound to carbon atoms were calculated in ideal positions with isotropic displacement parameters set to 1.2xUeq of the attached atom (1.5xUeq for methyl hydrogen atoms). The hydrogen atoms bound to the pyrrole

nitrogen atoms and the hydroxyl atoms of the phosphate ion were observed in a $\Delta$F map and refined with isotropic displacement parameters.

The function, $\Sigma w(|F_o|^2 - |F_c|^2)^2$, was minimized, where $w = 1/[(\sigma(F_o))^2 + (0.0516*P)^2 + (2.5503*P)]$ and $P = (|F_o|^2 + 2|F_c|^2)/3$. $R_w(F^2)$ refined to 0.124, with R(F) equal to 0.0500 and a goodness of fit, S, = 1.05. Definitions used for calculating R(F), $R_w(F^2)$ and the goodness of fit, S, are given below.[7] The data were checked for secondary extinction effects but no correction was necessary. Neutral atom scattering factors and values used to calculate the linear absorption coefficient are from the International Tables for X-ray Crystallography (1992).[8] All figures were generated using SHELXTL/PC.[9] Tables of positional and thermal parameters, bond lengths and angles, torsion angles and figures may be obtained from the CCDC by referencing CCDC number 1444560.

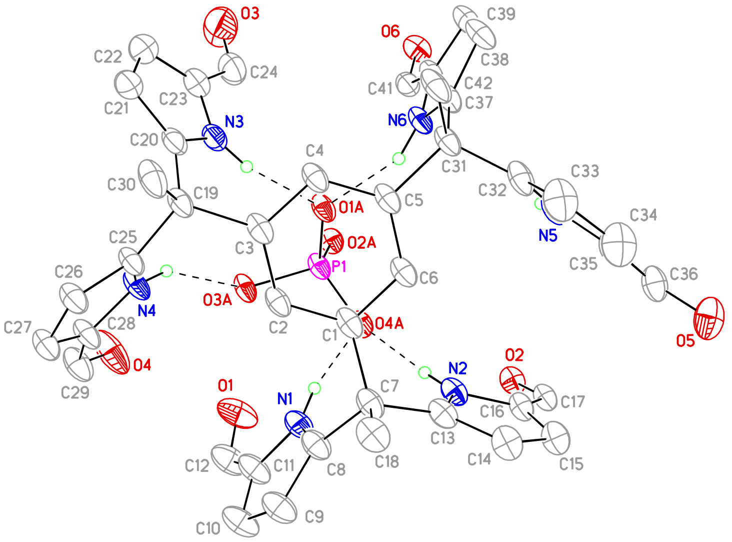


X-ray Experimental for **7b**·$TBAH_2PO_4$ (2 $C_{42}H_{36}N_6O_6$ - 2 $C_{16}H_{36}N^{1+}$ $H_2PO_4^{1-}$ - 3 $CHCl_3$): Crystals grew as thin, yellow plates by layering a solution of **7b**·$TBAH_2PO_4$ in $CHCl_3$ with *n*-pentane. The data crystal had approximate dimensions; 0.27 x 0.16 x 0.08 mm. The data were collected on an Agilent Technologies SuperNova Dual Source diffractometer using a $\mu$-focus Cu K$\alpha$ radiation source ($\lambda$ = 1.5418Å) with collimating mirror monochromators. A total of 1035 frames of data were collected using $\omega$-scans with a scan range of 0.5° and

a counting time of 14 seconds per frame using a detector offset of +/- 41.1° and a counting time of 38 seconds per frame using a detector offset of +/- 108.3°. The data were collected at 100 K using an Oxford Cryostream low temperature device. Details of crystal data, data collection and structure refinement are listed in Table ESI-6. Data collection, unit cell refinement and data reduction were performed using Agilent Technologies CrysAlisPro V 1.171.37.31.[12] The structure was solved by direct methods using SIR97[13] and refined by full-matrix least-squares on $F^2$ with anisotropic displacement parameters for the non-H atoms using SHELXL-2013.[4] Structure analysis was aided by use of the programs PLATON98[5] and WinGX.[6] The hydrogen atoms were calculated in ideal positions with isotropic displacement parameters set to 1.2xUeq of the attached atom (1.5xUeq for methyl hydrogen atoms.

The two tetra-n-butyl ammonium ions had highly anisotropic displacement parameters. One of the ions was refined with a disorder model. The disorder was modelled by assigning the variable x to the site occupancy for one component of the disorder and (1-x) to the site occupancy for the alternate component. The geometry of the two components was restrained by the extensive use of SADI instructions to maintain the C-C bonds and C-C-C angles to be equivalent. A common isotropic displacement parameter was assigned to the nitrogen atoms and the methylene carbon atoms while refining the variable x. A separate isotropic displacement parameter was refined for the methyl carbon atoms.

There are several regions of unidentified disordered solvent. The solvent was badly disordered and could not be reliably identified but was probably a mixture of the solvents used for crystallization, chloroform and n-hexane. The quantity and identity of the solvent in these voids was in doubt. The squeezed out solvent was not included in the UNIT card. The contributions to the scattering factors due to this solvent molecule were removed by use of the utility SQUEEZE[6] in PLATON98. PLATON98 was used as incorporated in WinGX.

The function, $\Sigma w(|F_o|^2 - |F_c|^2)^2$, was minimized, where $w = 1/[(\sigma(F_o))^2 + (0.1*P)^2]$ and $P = (|F_o|^2 + 2|F_c|^2)/3$. $R_w(F^2)$ refined to 0.345, with R(F) equal to 0.126 and a goodness of fit, S, = 1.61. Definitions used for calculating R(F),

$R_w(F^2)$ and the goodness of fit, S, are given below.[7] The data were checked for secondary extinction effects but no correction was necessary. Neutral atom scattering factors and values used to calculate the linear absorption coefficient are from the International Tables for X-ray Crystallography (1992).[8] All figures were generated using SHELXTL/PC.[9] Tables of positional and thermal parameters, bond lengths and angles, torsion angles and figures may be obtained from the CCDC by referencing CCDC number 1444561.

## Structure Refinement Parameters

**Table ESI-2** Crystal data and structure refinement for **6b**·2DMF.

| | | |
|---|---|---|
| CCDC number | 1444557 | |
| Empirical formula | C40 H44 Fe N6 O6 | |
| Formula weight | 760.66 | |
| Temperature | 133(2) K | |
| Wavelength | 0.71073 Å | |
| Crystal system | monoclinic | |
| Space group | P 21/c | |
| Unit cell dimensions | a = 12.5879(6) Å | $\alpha$ = 90°. |
| | b = 20.8699(10) Å | $\beta$ = 93.531(4)°. |
| | c = 13.6448(8) Å | $\gamma$ = 90°. |
| Volume | 3577.8(3) $Å^3$ | |
| Z | 4 | |
| Density (calculated) | 1.412 $Mg/m^3$ | |
| Absorption coefficient | 0.480 $mm^{-1}$ | |
| F(000) | 1600 | |
| Crystal size | 0.27 x 0.20 x 0.09 mm | |
| Theta range for data collection | 1.621 to 27.595°. | |
| Index ranges | -16<=h<=16, -27<=k<=27, -17<=l<=17 | |
| Reflections collected | 87367 | |
| Independent reflections | 8232 [R(int) = 0.1341] | |
| Completeness to theta = 25.242° | 99.8 % | |
| Absorption correction | Semi-empirical from equivalents | |
| Max. and min. transmission | 1.00 and 0.867 | |
| Refinement method | Full-matrix least-squares on $F^2$ | |
| Data / restraints / parameters | 8232 / 0 / 500 | |
| Goodness-of-fit on $F^2$ | 1.013 | |
| Final R indices [I>2sigma(I)] | R1 = 0.0471, wR2 = 0.0897 | |
| R indices (all data) | R1 = 0.1007, wR2 = 0.1109 | |
| Extinction coefficient | n/a | |
| Largest diff. peak and hole | 0.382 and -0.331 e.Å-3 | |

**Table ESI-3** Crystal data and structure refinement for **7b**·$2CHCl_3$.

| | | |
|---|---|---|
| CCDC number | 1444558 | |
| Empirical formula | C44 H38 Cl6 N6 O6 | |
| Formula weight | 959.50 | |
| Temperature | 100(2) K | |
| Wavelength | 0.71073 Å | |
| Crystal system | triclinic | |
| Space group | P -1 | |
| Unit cell dimensions | a = 10.1932(19) Å | $\alpha$ = 72.773(4)°. |
| | b = 12.318(2) Å | $\beta$ = 77.793(4)°. |
| | c = 19.654(4) Å | $\gamma$ = 81.490(4)°. |
| Volume | 2294.1(7) $Å^3$ | |
| Z | 2 | |
| Density (calculated) | 1.389 $Mg/m^3$ | |
| Absorption coefficient | 0.428 $mm^{-1}$ | |
| F(000) | 988 | |
| Crystal size | 0.300 x 0.230 x 0.150 mm | |
| Theta range for data collection | 3.059 to 27.483°. | |
| Index ranges | -13<=h<=13, -15<=k<=15, -25<=l<=25 | |
| Reflections collected | 23595 | |
| Independent reflections | 10438 [R(int) = 0.0717] | |
| Completeness to theta = 25.242° | 99.8 % | |
| Absorption correction | Semi-empirical from equivalents | |
| Max. and min. transmission | 1.00 and 0.654 | |
| Refinement method | Full-matrix least-squares on $F^2$ | |
| Data / restraints / parameters | 10438 / 81 / 600 | |
| Goodness-of-fit on $F^2$ | 1.140 | |
| Final R indices [I>2sigma(I)] | R1 = 0.0937, wR2 = 0.2330 | |
| R indices (all data) | R1 = 0.1450, wR2 = 0.2589 | |
| Extinction coefficient | n/a | |
| Largest diff. peak and hole | 0.945 and -1.034 e.$Å^{-3}$ | |

**Table ESI-4** Crystal data and structure refinement for **3b**·$(TBA)_2H_2P_2O_7$.

| | | |
|---|---|---|
| CCDC number | 1444559 | |
| Empirical formula | C62.50 H99 Cl N6 O11 P2 | |
| Formula weight | 1207.86 | |
| Temperature | 100(2) K | |
| Wavelength | 1.54184 Å | |
| Crystal system | monoclinic | |
| Space group | P 21/n | |
| Unit cell dimensions | a = 19.5077(3) Å | $\alpha$ = 90°. |
| | b = 15.3419(3) Å | $\beta$ = 95.4010(10)°. |
| | c = 22.1726(3) Å | $\gamma$ = 90°. |
| Volume | 6606.47(19) $Å^3$ | |
| Z | 4 | |
| Density (calculated) | 1.214 $Mg/m^3$ | |
| Absorption coefficient | 1.457 $mm^{-1}$ | |
| F(000) | 2604 | |
| Crystal size | 0.160 x 0.110 x 0.060 $mm^3$ | |
| Theta range for data collection | 2.886 to 74.239°. | |
| Index ranges | -23<=h<=24, -17<=k<=18, -26<=l<=27 | |
| Reflections collected | 35854 | |
| Independent reflections | 13076 [R(int) = 0.0339] | |
| Completeness to theta = 67.684° | 99.8 % | |
| Absorption correction | Semi-empirical from equivalents | |
| Max. and min. transmission | 1.00 and 0.961 | |
| Refinement method | Full-matrix least-squares on $F^2$ | |
| Data / restraints / parameters | 13076 / 535 / 761 | |
| Goodness-of-fit on $F^2$ | 1.029 | |
| Final R indices [I>2sigma(I)] | R1 = 0.0603, wR2 = 0.1574 | |
| R indices (all data) | R1 = 0.0777, wR2 = 0.1710 | |
| Extinction coefficient | n/a | |
| Largest diff. peak and hole | 1.034 and -0.466 e.$Å^{-3}$ | |

**Table ESI-5** Crystal data and structure refinement parameters for **4b·**$TBAH_2PO_4$.

| | | |
|---|---|---|
| CCDC number | 1444560 | |
| Empirical formula | C46.50 H64.50 Cl1.50 N5 O8 P | |
| Formula weight | 905.67 | |
| Temperature | 100(2) K | |
| Wavelength | 1.54184 Å | |
| Crystal system | triclinic | |
| Space group | P -1 | |
| Unit cell dimensions | a = 9.9599(4) Å | α = 81.050(3)°. |
| | b = 14.6932(6) Å | β = 75.273(3)°. |
| | c = 16.4802(5) Å | γ = 88.017(3)°. |
| Volume | 2304.10(15) $Å^3$ | |
| Z | 2 | |
| Density (calculated) | 1.305 $Mg/m^3$ | |
| Absorption coefficient | 1.802 $mm^{-1}$ | |
| F(000) | 966 | |
| Crystal size | 0.170 x 0.070 x 0.050 $mm^3$ | |
| Theta range for data collection | 2.805 to 74.533°. | |
| Index ranges | -12<=h<=12, -16<=k<=18, -20<=l<=19 | |
| Reflections collected | 24272 | |
| Independent reflections | 9074 [R(int) = 0.0371] | |
| Completeness to theta = 67.684° | 99.5 % | |
| Absorption correction | Semi-empirical from equivalents | |
| Max. and min. transmission | 1.00 and 0.863 | |
| Refinement method | Full-matrix least-squares on $F^2$ | |
| Data / restraints / parameters | 9074 / 6 / 607 | |
| Goodness-of-fit on $F^2$ | 1.047 | |
| Final R indices [I>2sigma(I)] | R1 = 0.0500, wR2 = 0.1188 | |
| R indices (all data) | R1 = 0.0641, wR2 = 0.1237 | |
| Extinction coefficient | n/a | |
| Largest diff. peak and hole | 0.749 and -0.617 e.$Å^{-3}$ | |

**Table ESI-6.** Crystal data and structure refinement parameters for [**7b**·$TBAH_2PO_4$]$_2$.

| | | |
|---|---|---|
| CCDC number | 1444561 | |
| Empirical formula | C119 H151 Cl9 N14 O20 P2 | |
| Formula weight | 2478.52 | |
| Temperature | 100(2) K | |
| Wavelength | 1.54184 Å | |
| Crystal system | triclinic | |
| Space group | P -1 | |
| Unit cell dimensions | a = 16.0755(7) Å | α = 93.415(2)°. |
| | b = 18.9299(7) Å | β = 96.103(3)°. |
| | c = 24.4976(6) Å | γ = 111.261(4)°. |
| Volume | 6869.2(5) $Å^3$ | |
| Z | 2 | |
| Density (calculated) | 1.198 $Mg/m^3$ | |
| Absorption coefficient | 2.423 $mm^{-1}$ | |
| F(000) | 2612 | |
| Crystal size | 0.270 x 0.160 x 0.080 $mm^3$ | |
| Theta range for data collection | 2.950 to 74.200°. | |
| Index ranges | -13<=h<=19, -23<=k<=22, -27<=l<=30 | |
| Reflections collected | 40560 | |
| Independent reflections | 26292 [R(int) = 0.0646] | |
| Completeness to theta = 67.684° | 98.1 % | |
| Absorption correction | Semi-empirical from equivalents | |
| Max. and min. transmission | 1.00 and 0.560 | |
| Refinement method | Full-matrix least-squares on $F^2$ | |
| Data / restraints / parameters | 26292 / 3337 / 1638 | |
| Goodness-of-fit on $F^2$ | 1.544 | |
| Final R indices [I>2sigma(I)] | R1 = 0.1255, wR2 = 0.3052 | |
| R indices (all data) | R1 = 0.1811, wR2 = 0.3446 | |
| Extinction coefficient | n/a | |
| Largest diff. peak and hole | 1.140 and -1.260 e.$Å^{-3}$ | |

## $^1$H and $^{13}$C NMR Spectra

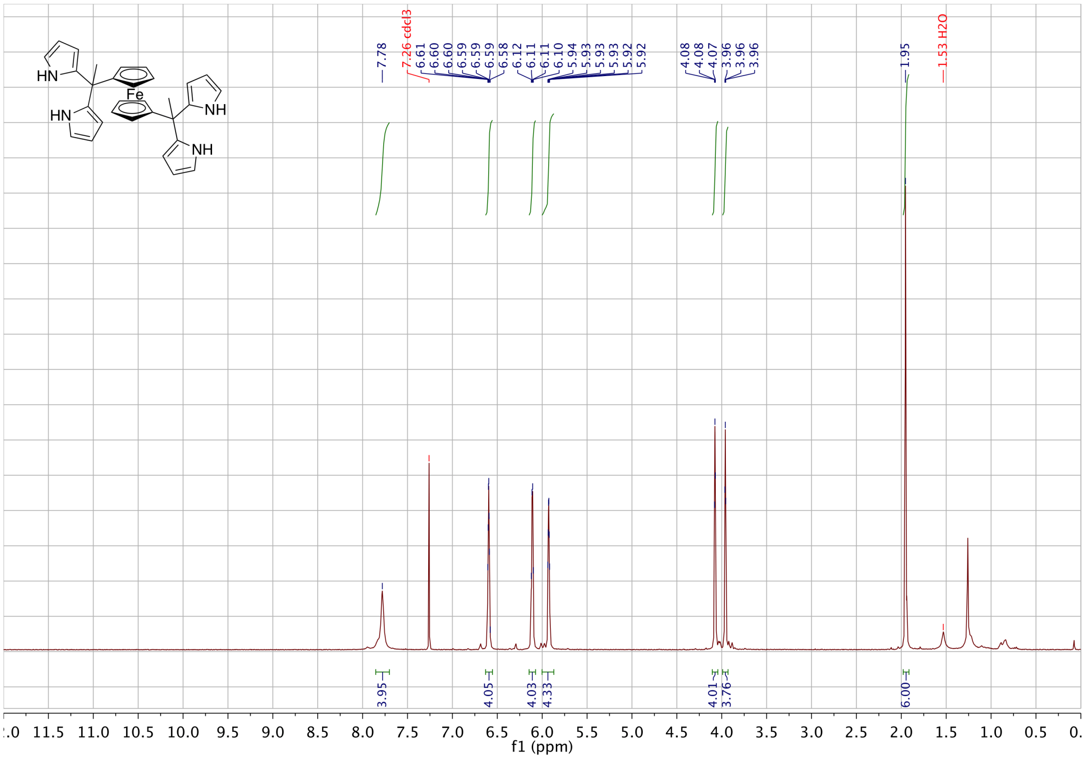

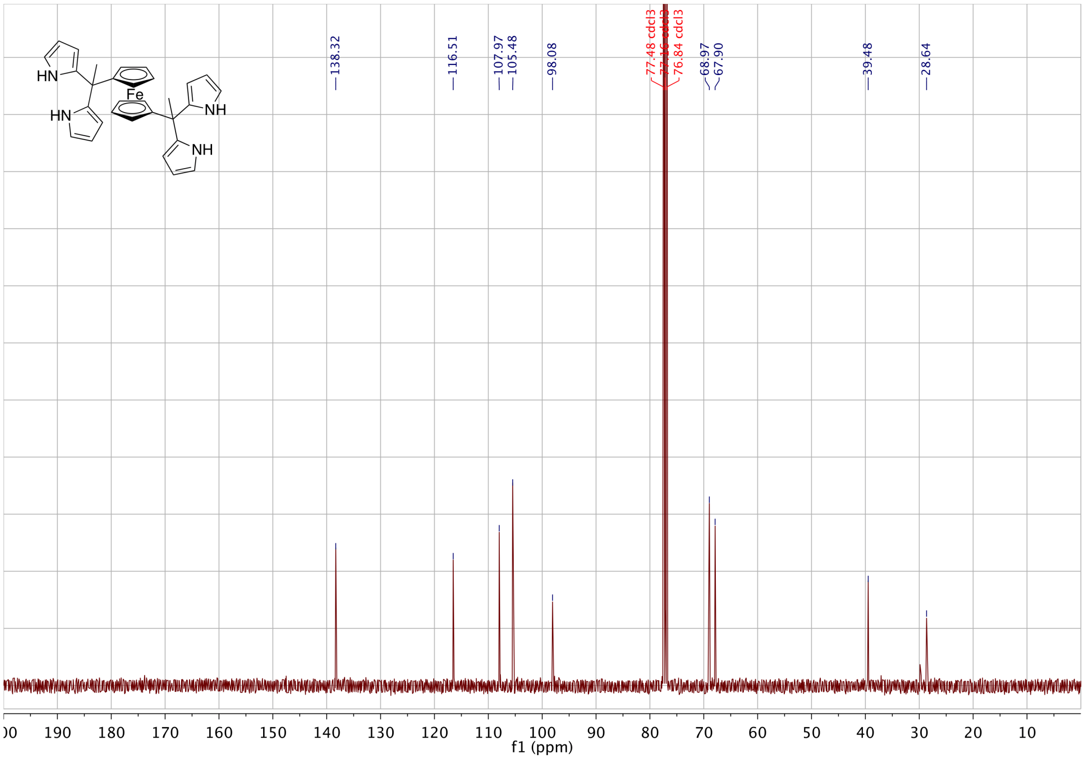

**Fig. SI-23** Top: $^1$H NMR (400 MHz) Bottom: $^{13}$C NMR (100 MHz) spectra of **6a** in $CDCl_3$.

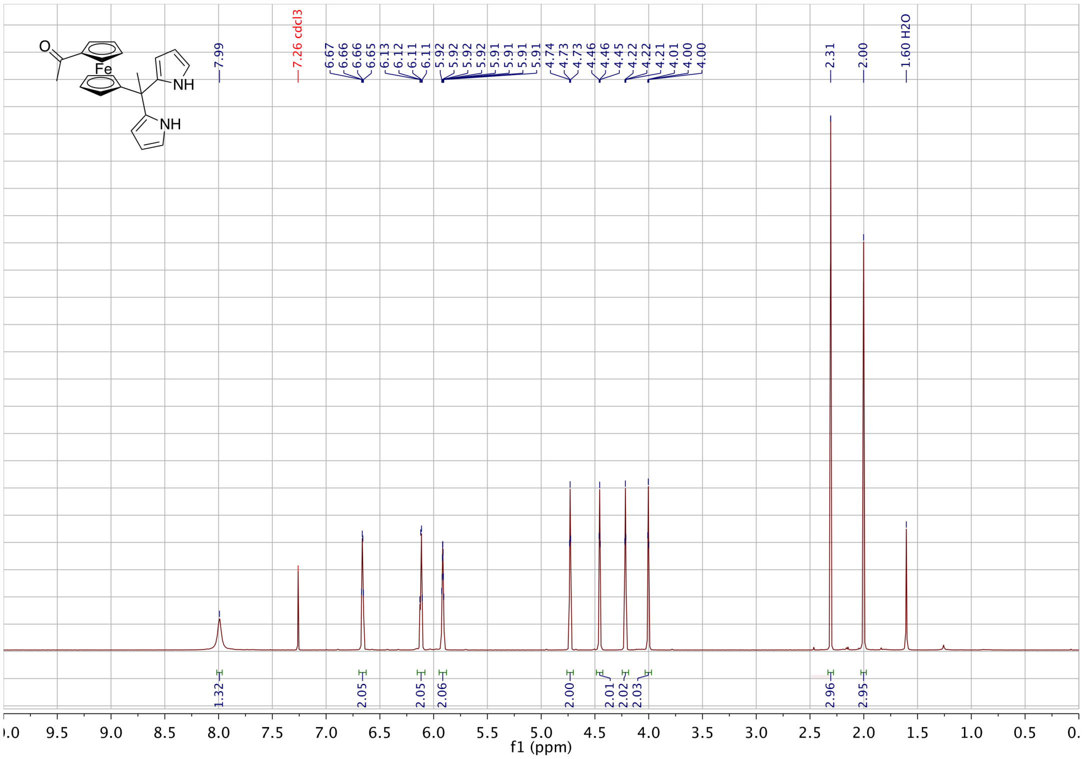

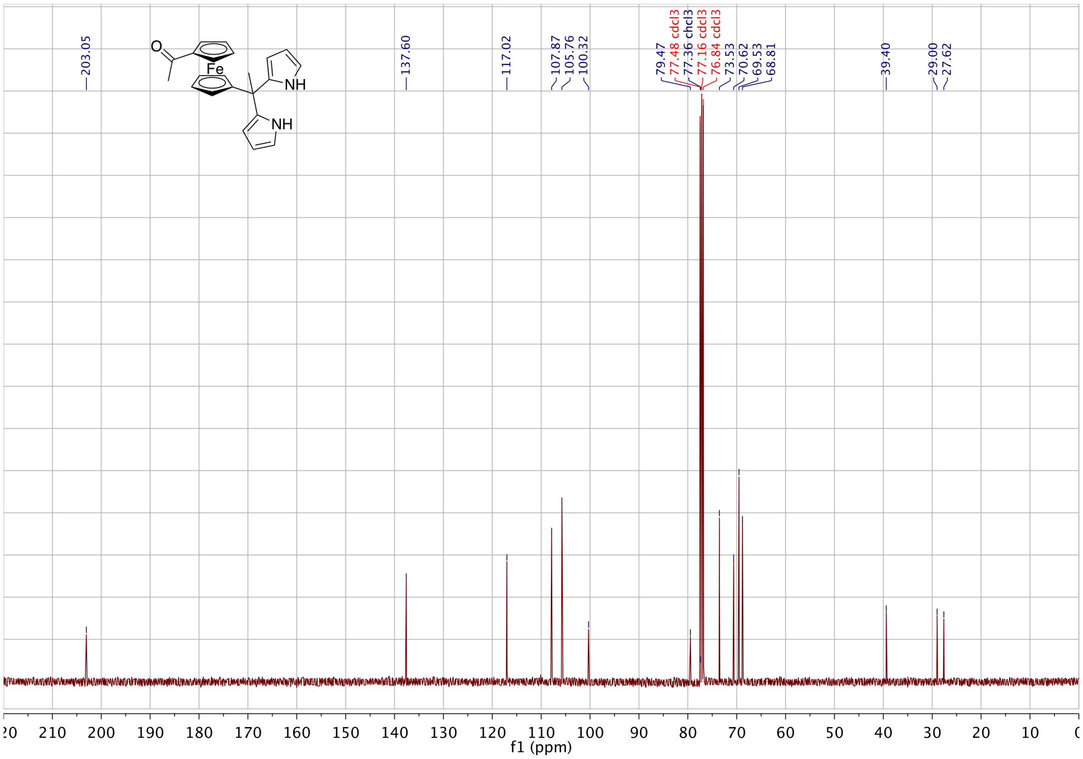

**Fig. SI-24** Top: $^{1}H$ NMR (400 MHz) Bottom: $^{13}C$ NMR (100 MHz) spectra of **6a'** in $CDCl_3$.

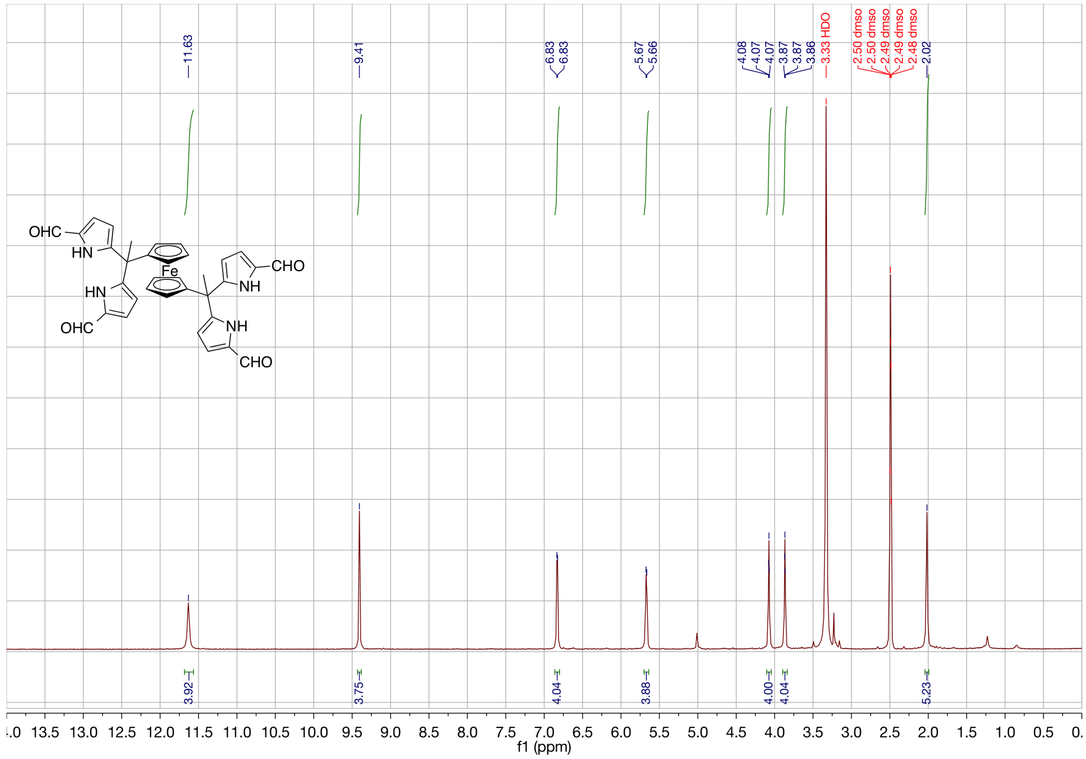


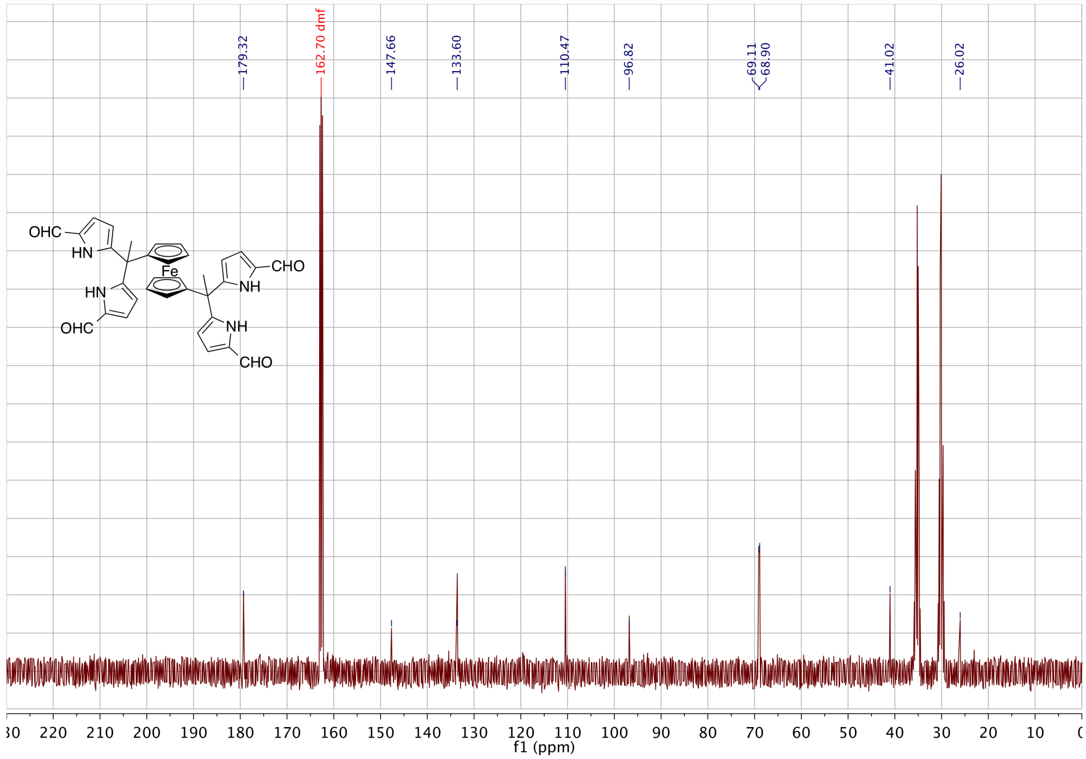


**Fig. SI-25** Top: $^{1}H$ NMR (400 MHz) spectrum of **6b** in DMSO-$d_6$. Bottom: $^{13}C$ NMR (100 MHz) spectrum of **6b** in DMF-$d_7$.

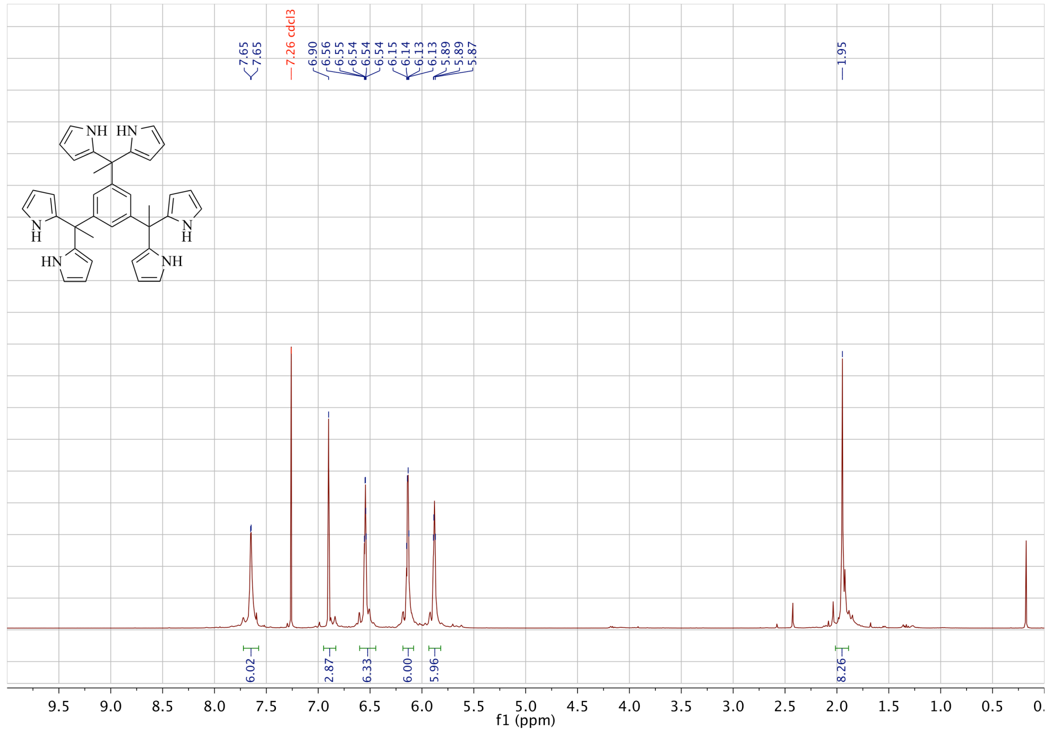


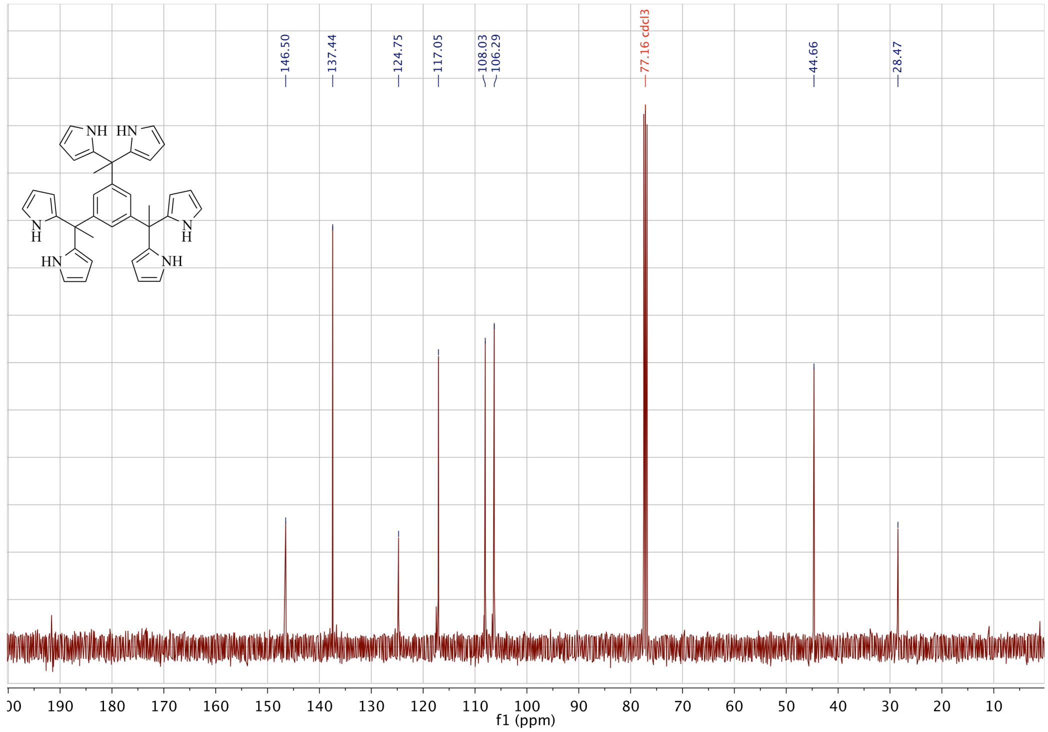


**Fig. SI-26** Top: $^{1}$H NMR (400 MHz) Bottom: $^{13}$C NMR (100 MHz) spectra of **7a** in $CDCl_3$.

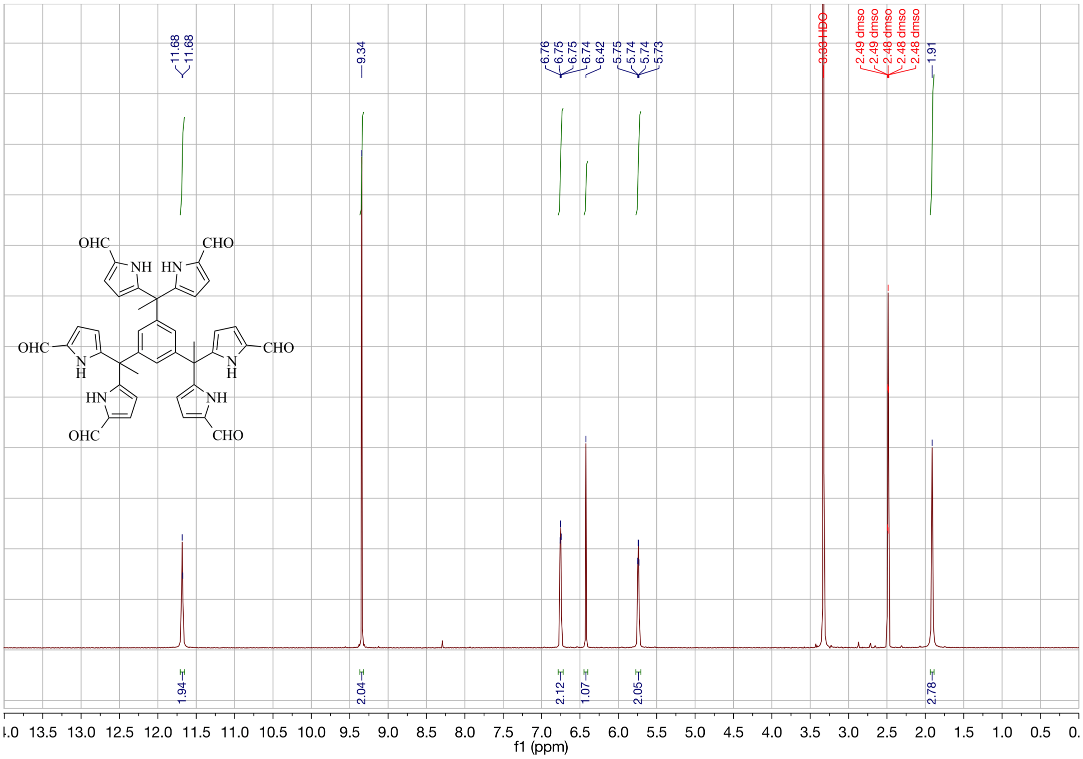


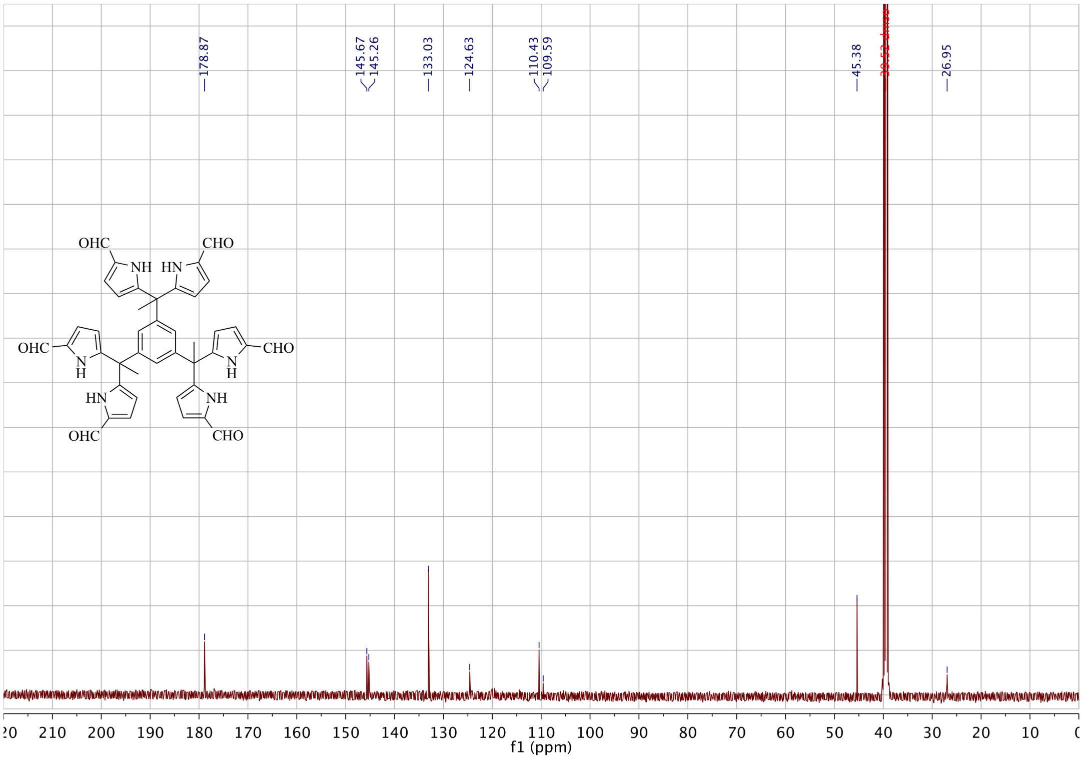


**Fig. SI-27** Top: $^{1}$H NMR (400 MHz), bottom: $^{13}$C NMR (100 MHz) spectra of **7b** in DMSO-$d_6$.

### Notes and References


1. M. K. Deliomeroglu, V. M. Lynch and J. L. Sessler, *Chem. Commun.*, 2014, **50**, 11863–11866.
2. SAINT V8.27B Bruker AXS Inc, Madison, WI, 2012.
3. L. Palatinus and G. Chapuis, *J. Appl. Crystallogr.*, 2007, **40**, 786–790.
4. G. M. Sheldrick, SHELXL-2013 (Program for the Refinement of Crystal Structures), *Acta Cryst.*, 2008, **A64**, 112–122.
5. A. L. Spek, *PLATON, A Multipurpose Crystallographic Tool*, Utrecht University, The Netherlands, 1998.
6. L. J. Farrugia, *J. Appl. Crystallogr.*, 1999, **32**, 837–838.
7. $R_w(F^2) = \{\Sigma w(|F_o|^2 - |F_c|^2)^2/\Sigma w(|F_o|)^4\}^{1/2}$ where w is the weight given each reflection.

   $R(F) = \Sigma(|F_o| - |F_c|)/\Sigma|F_o|\}$ for reflections with $F_o > 4(\sigma(F_o))$.

   $S = [\Sigma w(|F_o|^2 - |F_c|^2)^2/(n - p)]^{1/2}$, where n is the number of reflections and p is the number of refined parameters.
8. A. J. C. Wilson (editor), *International Tables for X-ray Crystallography. Vol. C, Tables 4.2.6.8 and 6.1.1.4*, Boston: Kluwer Academic Press, 1992.
9. G. M. Sheldrick, *SHELXTL/PC (Version 5.03)*, Siemens Analytical X-ray Instruments, Inc., Madison, Wisconsin, USA, 1994.
10. CrystalClear 1.40, Rigaku Americas Corporation, The Woodlands, TX, 2008.
11. A. Altomare, M.C. Burla, M. Caliandro, M. Camalli, B. Carrozzini, G.L. Cascarano, L. De Caro, C. Giacovazzo, G. Polidori and R. Spagna, SIR2004, A program for crystal structure solution, *J. Appl. Crystallogr.*, 2005, **38**, 381–388.
12. CrysAlisPro. Agilent Technologies, Agilent Technologies UK Ltd., Oxford, UK, SuperNova CCD System, CrysAlicPro Software System, 1.171.37.31, 2013.
13. A. Altomare, M. C. Burla, M. Camalli, G. L. Cascarano, C. Giacovazzo, A. Guagliardi, A. G. Moliterni, G. Polidori and R. Spagna, *J. Appl. Crystallogr.*, 1999, **32**, 115–119.
14. P. V. D. Sluis and A. L. Spek, SQUEEZE. *Acta Cryst.*, 1990, **A46**, 194–201.

15. L. Palatinus and G. Chapuis, *J. Appl. Crystallogr.*, 2007, **40**, 786–790.
